# The perfect storm: unraveling the interplay of genetic predisposition and viral triggers in type 1 diabetes pathogenesis

**DOI:** 10.3389/fendo.2025.1734431

**Published:** 2025-12-17

**Authors:** Daniel A. Veronese-Paniagua, Jeffrey R. Millman

**Affiliations:** 1 Roy and Diana Vagelos Division of Biology and Biomedical Sciences, Washington University School of Medicine, St. Louis, MO, United States; 2 Division of Endocrinology, Metabolism, and Lipid Research, Washington University School of Medicine, St. Louis, MO, United States

**Keywords:** autoimmunity, coxsackievirus B (CVB), enterovirus, HLA, immune tolerance, stem cell-derived islets, type I interferon (IFN), β cells

## Abstract

Type 1 diabetes (T1D) is a chronic autoimmune disease characterized by the T cell-mediated destruction of insulin-secreting pancreatic β cells, leaving patients reliant on exogenous insulin to establish normoglycemia. Despite advancements in glucose management, the precise pathological mechanisms linking genetic predisposition and environmental triggers to the loss of immune tolerance remain incompletely understood, hindering the development of preventative therapies. This comprehensive review synthesizes clinical, experimental, and epidemiological data to detail the intricate pathogenesis of T1D, focusing on the convergence of autoimmunity, high-risk genetics, and enteroviral infection. We discuss how T1D is fundamentally a disease caused by failures in central and peripheral tolerance mechanisms, leading to the activation and infiltration of autoreactive CD4+ and CD8+ T cells into the pancreatic islets. We then explore the profound influence of genetic susceptibility, highlighting the role of HLA and non-HLA genes (e.g., *IFIH1*, *TYK2*) that modulate innate immune response, connecting genetic susceptibility to the pro-inflammatory response to pathogens. We also discuss enterovirus infection, particularly by coxsackievirus B (CVB), and its potential role as a critical environmental trigger. We demonstrate how CVB utilizes and subverts host cellular machinery to promote β cell stress and facilitate immune evasion, as well as evidence for its ability to establish a persistent low-grade infection within the pancreas. Finally, we emphasize the indispensable role of advanced human models, such as human pluripotent stem cell-derived islets. Elucidating the precise mechanisms linking genetic and viral risk factors in human-relevant contexts is critical. Future research must prioritize addressing these knowledge gaps to develop targeted, preemptive interventions that can successfully delay or prevent T1D onset.

## 1 Introduction

Diabetes mellitus is a chronic condition marked by high blood glucose levels caused by the dysfunction and/or death of pancreatic insulin-secreting β cells. Type 1 diabetes (T1D), specifically, is an autoimmune disorder marked by little to no insulin production and secretion due to the attack and loss of β cells by autoreactive T cells. This disease occurs in about 8–20 per 100,000 people, affecting approximately 27–54 million people worldwide (1). All T1D patients rely on daily glucose monitoring and exogenous insulin injections to regulate blood glucose levels. However, insulin administration is not a cure and fails to mimic the precise glycemic regulation provided by the pancreatic islets of Langerhans, which are composed of endocrine cells including β, glucagon-secreting α, somatostatin-secreting δ, and pancreatic polypeptide-secreting γ cells. Thus, patients can still experience hyper- and hypoglycemic episodes due to miscalculated amounts of injected insulin prior to meals and exercise (2, 3). Additionally, T1D can lead to multiple comorbidities long-term, such as retinopathy, nephropathy, neuropathy, and cardiovascular disease (4, 5). These complications decrease the quality of life and increase the mortality rate of T1D patients whilst also increasing healthcare care costs. The deficit of proper therapeutics to protect β cells and prevent T1D exists because of our incomplete understanding of the mechanisms behind the complex initiation and progression of the disease.

**Figure 1:**
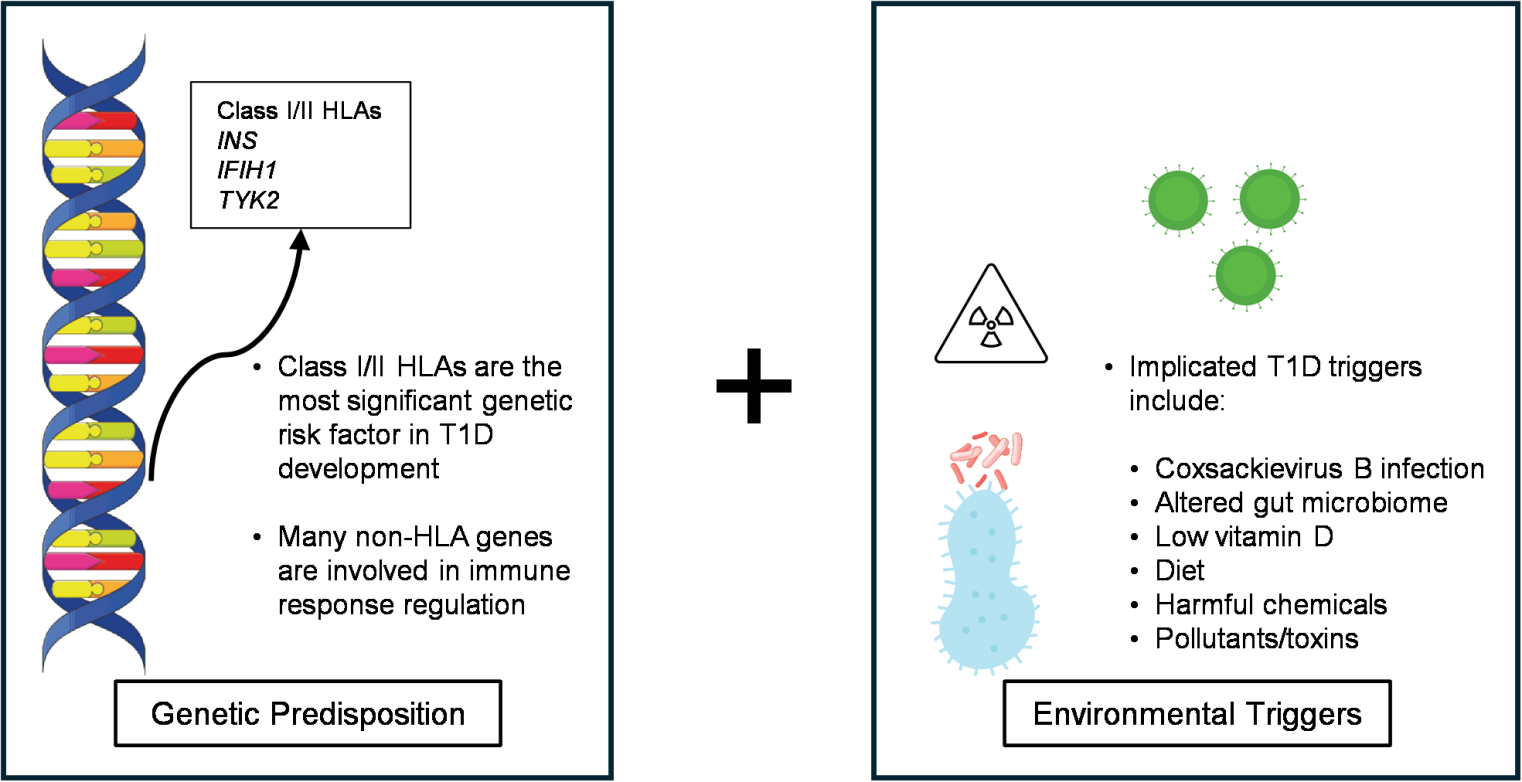
Schematic showing the convergence of genetic predisposition and environmental triggers in T1D pathogenesis.

Studies have found that the best predictor for islet autoimmunity is genetic background, with predisposition caused by genetic susceptibility in multiple loci, including *INS* and the class I and II Major Histocompatibility Complex (MHC)/human leukocyte antigen (HLA) genes (**Figure 1**) (6, 7). However, genetic background alone cannot explain the 35% discordant rate for T1D among monozygotic twins by age 60 nor the doubling of the annual incidence rate in the last twenty years to an estimated 3.9% in the United States (6, 8, 9). Multiple groups have instead turned to environmental factors as a trigger for β cell autoimmunity. This notion is supported by increased T1D incidence amongst children from genetically stable populations and in people from low-incidence countries who migrated to high-incidence countries (10). In humans, many studies have suggested enterovirus infection, particularly by coxsackievirus B (CVB), as a potential pathogenic trigger (**Figure 1**). A study found that coxsackieviruses composed 64% of the enteroviruses found in excess in the stool of T1D susceptible children up to one year prior to the first detection of islet autoantibodies (AAb) (11). In Non-Obese Diabetic (NOD) mice, a murine model of T1D, vaccination against CVB serotypes 1–6 protected mice from CVB-induced T1D onset (12). However, the mechanisms governing how CVB infection may trigger T1D remain unknown.

**Figure 2:**
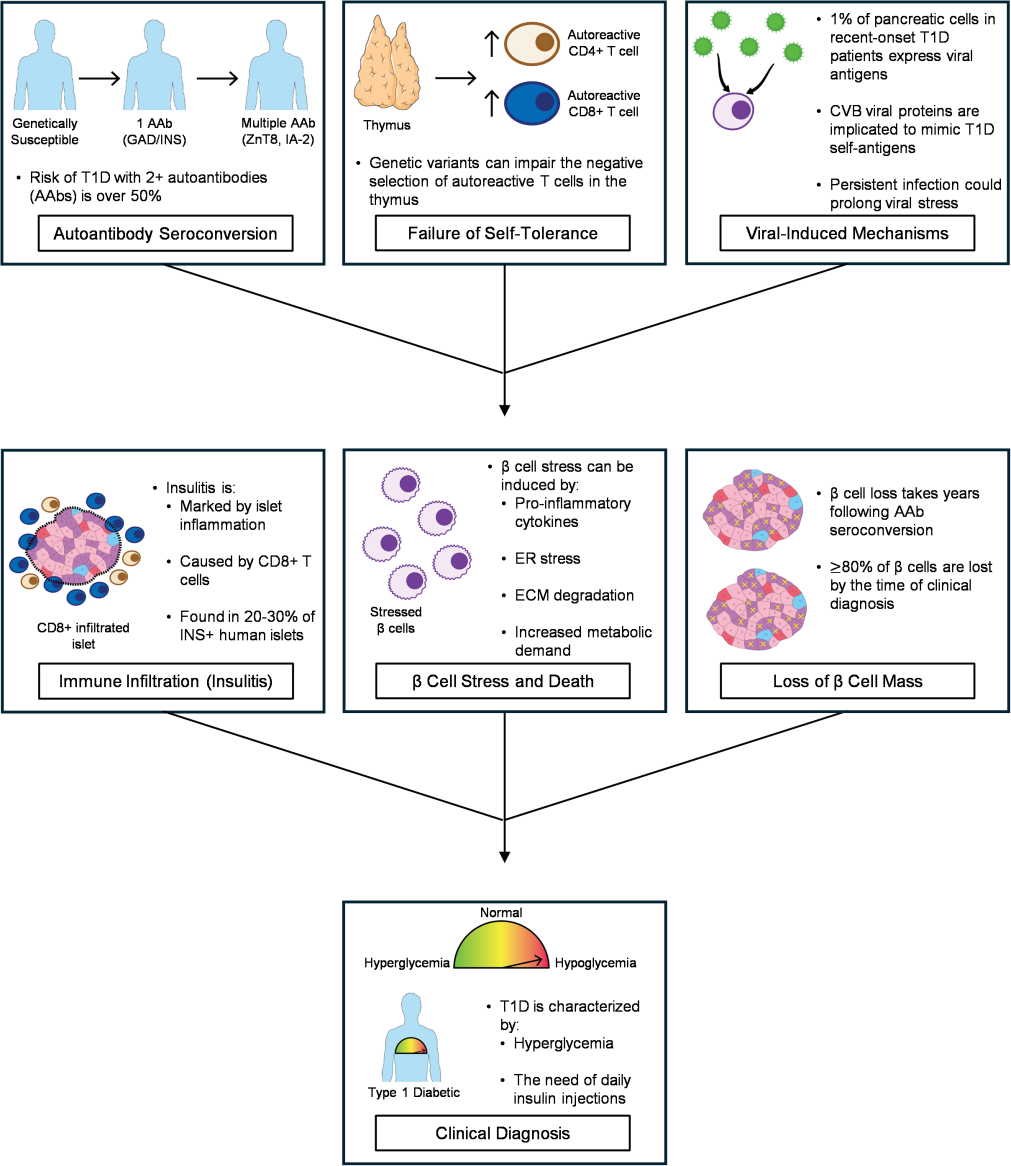
Schematic illustrating T1D initiation and progression as well as the potential role of viral infections as a trigger.

The precise pathological cause of T1D remains undetermined; however, the first stage of the disease is characterized by the presence of AAb, normoglycemia, and β cell autoimmunity (13). In most patients, the first AAb is either against insulin or glutamic acid decarboxylase (GAD) (14). By the second stage, the patient remains asymptomatic but has developed AAb against other antigens, including zinc transporter 8 (ZnT8) and insulinoma-associated antigen-2 (IA-2) (13–19). The presence of two or more AAb is associated with an over 50% chance of progression to clinical diagnosis (**Figure 2**) (14–18). The third stage is marked by significant loss of β cell mass and clinical diagnosis of T1D (13). Although these AAb are normally used as biomarkers, there has been no direct evidence for a causative role, with 71-73% of individuals with a single AAb at seroconversion remaining T1D free (14, 15, 19).

While the field has taken great strides to understand T1D onset, the complexity of this disease has presented persistent challenges, particularly regarding the interplay between genetic predisposition and environmental triggers. Here, we will provide a comprehensive overview of the intricate relationship between T1D, the immune system, and genetic background, as well as enteroviral infections as a potential environmental trigger, drawing upon clinical, experimental, and epidemiological data. We will also highlight emerging model systems and technologies that have helped dissect the influence of these factors. The objective of this review is to outline our current understanding of T1D pathogenesis and highlight significant knowledge gaps, thereby motivating future studies to improve our understanding of the precise mechanisms linking genetic and viral risk factors in the context of T1D. Ultimately, elucidating these will be crucial for developing novel therapies to delay or prevent T1D in genetically susceptible individuals.

## 2 T1D as an autoimmune disease marked by a breakdown of central and peripheral tolerance and activation of autoreactive T cells

Studies quantifying the link between genetic predisposition associated with T1D and cell-type-specific transcriptional signatures have shown that immune cells are the cell type most associated with T1D (20). Specifically, the immune attack leading to β cell death is largely mediated by autoreactive CD4+ and CD8+ T cells. Clinical trials using immunosuppressants against these cells have preserved C-peptide and reduced HbA1c in patients with recent-onset T1D (21–23). The presence of these autoreactive T cells points to a fundamental failure in the mechanisms of central and peripheral tolerance, a process that originates in the thymus gland, the primary site of T cell ontogeny.

### 2.1 The critical role of the thymus gland and regulatory T cells in autoimmunity

In healthy individuals, the thymus plays a critical regulatory role in preventing various autoimmune diseases (24). It achieves this by negatively selecting more than 95% of immature T lymphocytes, including potentially autoreactive cells, thereby defining the pool of T cells that respond to only non-self-antigens (25, 26). During this process, thymocytes are probed to recognize host MHC molecules by presenting peptides that originate from self-antigens (26). Lymphocytes with insufficient affinity undergo cell death, while cells with too strong an affinity are later eliminated through negative selection (26). Any T cells that escape this rigorous thymic negative selection are then targeted by regulatory T cells (Tregs), which act as a crucial secondary checkpoint (25, 26). In T1D, thymus-dependent tolerance and Treg function are thought to become dysfunctional, leading to the retention of autoreactive T cells that attack pancreatic islets (**Figure 2**). It is speculated that SARS-CoV-2 infection could exacerbate this mechanism by depleting Treg cells or diminishing their function due to suppression of FoxP3 (27). Despite this understanding, the precise mechanisms and timeline of autoreactive T cell dysregulation in humans are not fully understood because of the scarcity of samples from genetically susceptible, nondiabetic individuals. This gap highlights the critical need for longitudinal sample acquisition from these individuals across various stages prior to clinical onset to better delineate the peripheral events driving T1D pathogenesis.

### 2.2 Autoreactive T cell activation and infiltration in the pancreas

Beyond the failures of central and peripheral tolerance, the progression of T1D relies on the precise mechanisms by which autoreactive T cells are presented with self-antigens in the periphery. In T1D, autoreactive T cells develop in the pancreatic lymph nodes, where dendritic cells and, eventually, B cells, serve as antigen-presenting cells (APCs) to CD4+ T cells via MHC class II proteins (19, 28, 29). CD4+ T cells are presented preproinsulin epitopes by different alleles of either class II HLA-DR or -DQ, with a trend toward dominant restriction to HLA-DQ (30). Once activated, CD4+ T cells modulate the cascade of immune response by secreting cytokines and molecules. Secretion of pro-inflammatory molecules by CD4+ T cells—as observed in T1D, vaccine, and tuberculosis models—activates and increases the proliferation, survival, and cytotoxic ability of CD8+ T cells (31–33). The combined activation of CD4+ and CD8+ T cells in the pancreatic lymph nodes prepares these cells for the subsequent migration into the pancreas, where they ultimately destroy the β cells.

Although autoreactive T cells are a hallmark of T1D, preproinsulin-specific CD8+ T cells are present at comparable levels in the exocrine pancreas of healthy, AAb+, and T1D patients (30, 34). These observations suggest CD8+ T cells are initially monospecific and reside in the pancreas as a default state, only becoming multi-specific and infiltrating the islet as pathogenesis advances. Pseudotime analysis of tissue samples from healthy, AAb+, and T1D patients has shown a heterogenous activation profile of autoreactive CD8+ with activation profiles enriched in islet-infiltrated cells independent of other immune cell types (35). Other studies have shown the composition of antigen-specific CD8+ T cells differs across patients, with T1D patients exhibiting higher percentages of memory T cell subtypes, while healthy control populations are mostly compromised of naïve T cells (36, 37). A substantial increase in the proportion of resident memory T cells in recent-onset T1D islets suggests a significant role for these cells, potentially triggered by an infectious agent (36). Stem cell-like CD8+TCF1^high^ autoreactive memory T cells have also been reported in the pancreatic lymph nodes of NOD mice. These cells exhibited high Wnt signaling, expressed self-renewal genes, and were capable of differentiation into effector TCF1^low^CD8+ T cells that relocated into the pancreas (38). When transplanted into NOD SCID mice, as few as 20 of these CD8+TCF1^high^ stem-like T cells could induce T1D while as many as 100,000 TCF1^low^ cells did not (38). However, whether these events occur in human T1D pathogenesis remains unknown. Future studies could utilize patient-derived human pluripotent stem cells and differentiate them into T cells to decipher the mechanisms that trigger the population shift from naïve T cells to other cell types, including CD8+TCF1^high^ cells, and determine whether these autoreactive T cells can lead to the destruction of β cells using human *in vitro* models.

Similar proportions of islet-specific CD8+ T cells are found in the peripheral blood of healthy, AAb+, and diabetic patients; however, they are significantly enriched in the pancreas of AAb+ and T1D patients with no differences between pancreatic compartments (29, 30, 34, 36, 39). More specifically, the number and density of CD8+ T cells in close proximity to pancreatic islets are elevated in AAb+ and T1D patients and, as the disease progresses, further accumulation is observed (30, 34). β cells promote this process by the secretion of IFNα (18). In NOD mice, a T1D mouse model, class I MHC expression is necessary to present β cell autoantigens to CD8+ T cells for subsequent infiltration into the pancreas (40). This process is facilitated by endothelial cell-mediated insulin degradation and consequent presentation of insulin epitopes via class I MHC molecules to CD8+ T cells, allowing the T cells to adhere to the vasculature (40). The islets within AAb+ and diabetic patients have been shown to overexpress class I MHC/HLA molecules, as well as the components necessary for forming functional class I HLA complexes (18, 35, 41, 42). Future work is needed to validate endothelial cell-mediated T cell extravasation. These experiments should aim to use human pancreatic endothelial cells from T1D donors and autoreactive CD8+ T cells to perform *in vitro* transwell-based extravasation assays. Nevertheless, this intricate process of extravasation, mediated by various cellular and molecular signals, is a prerequisite for the T cells to infiltrate the islets and initiate the destructive phase of T1D.

To infiltrate the islet, T cells must trigger the cathepsin-mediated loss of peri-islet ECM to bypass the peri-islet membrane (43, 44). The degradation of the peri-islet membrane is a critical step that allows these accumulated T cells to finally gain access to the pancreatic islets and cause damage. Once infiltrated, the inflammatory microenvironment in the islet is marked by increased expression of cytokines related to inflammation and innate immune responses, including IFNγ, IL-18, IL-15, and IL-22, as well as chemokines and other ligands, such as CXCL1, CCL7, and CXCL9 (36, 45). These pro-inflammatory cytokines reduce β cell secretory function, insulin granule synthesis and content, gap junction coupling, and induce apoptosis via prolonged endoplasmic reticulum (ER) stress and inflammatory responses (18, 46). This pro-inflammatory islet environment is termed insulitis (**Figure 2**). Insulitis is classified as ≥15 CD45+ cells per islet in a minimum of three islets (47). Infiltrating cells can be found in the endocrine-exocrine interface (peri-insulitis) or within the islet parenchyma (intra-insulitis), but most autoreactive T cells are found in the periphery of islets (36, 47, 48). Regardless, insulitis marks the active phase of β cell destruction, where the invading immune cells directly and indirectly contribute to cell death.

In humans, roughly 20-30% of insulin-containing islets have been shown to exhibit insulitis compared to 2.9% in insulin-deficient islets (48, 49). CD8+ T cells are the predominant cell type, increasing with decreasing insulin-positive area but disappearing when insulin positivity is completely lost, despite exocrine infiltration staying the same (29, 49). However, insulitis is rare in nondiabetic, AAb+ individuals and only affects a small part of the islet (13, 49). Current datasets from cadaveric patients indicate that insulitis is mostly found in young, recent-onset patients and rarely in long-duration T1D patients (>1 year) (49). However, this observation is hard to support throughout disease progression of live patients since there are limited biopsy samples predating AAb+ and T1D onset (49). Ultimately, while immune-mediated β cell death is a process that unfolds over many years in humans, the limited data from cadaveric samples only offer us discrete snapshots, highlighting the persistent challenges in understanding the full spectrum of T1D pathogenesis. Overall, T1D is fundamentally a disease of immune tolerance failure, evidenced by the retention and subsequent activation of autoreactive CD8+ T cells that infiltrate the pancreas. These mechanistic changes set the stage for investigating how genetic factors modulate this tolerance breakdown and how environmental triggers initiate the peripheral activation cascade.

## 3 The genetic landscape of T1D: from risk scores to functional pathways

Predisposition to T1D is caused by genetic susceptibility in multiple loci, including *INS*, *IFIH1*, and the class I and II HLA genes (6). For most of the twentieth century, little was known about T1D disease progression prior to clinical onset. However, advances in genetic screens have allowed the creation of a T1D genetic risk score, which accounts for the presence of known at-risk genes in patients. This cumulative score can effectively distinguish healthy patients from T1D, type 2 diabetes, and monogenic forms of diabetes, such as maturity-onset diabetes of the young (50–53). Together with biometric values, the presence of certain single-nucleotide polymorphisms (SNPs) and haplotypes can even help predict which AAb will arise first. These T1D risk variants are frequently found in regions that control gene activity across various cell types, including those rooted in immune cells and the exocrine pancreas (20, 54–57). Therefore, these susceptibility genes are not just markers but active players that contribute to the autoimmune response in T1D patients.

### 3.1 T1D-associated genes: impact on immune function

Approximately 40-50% of the genetic risk of developing T1D is attributed to the class I and II MHC/HLA region, with class II being the most significant, as it is essential to the adaptive immune system (**Figure 1**) (7, 13, 50). Typically, APCs present external antigens to CD4+ T cells via class II HLA molecules. These molecules are composed of heterodimers, which are genetically determined by genes located at the *HLA-DR*, *HLA-DQ*, and *HLA-DP* loci (13). The presence of particular variants within each of the three loci can increase or decrease the risk of developing T1D. For instance, patients carrying DRB1*04XX-DQA1*0301-DQB1*0302 and DRB1*0301-DQA1*0501-DQB1*0201 haplotypes are the most susceptible to developing T1D (58, 59). Studies have also shown that heterozygosity in these loci, such as DRB1*03-DQB1*02/DRB1*0401-DQB1*0302, make it at least six times more likely to develop T1D compared to homozygosity for either haplotype (52). This increased risk is due to the formation of highly susceptible trans-encoded DQ heterodimers (58, 60). In contrast, DRB1*1501-DQA1*0102-DQB1*0602 confers protection against T1D (58). The *HLA-DPB1* locus also contributes to risk, though its effect is less profound than the *DR* and *DQ* loci, with DPB1*0301 and DPB1*0202 associated with risk and DPB1*0402 acting as a protective allele in DR3 carrying patients (61). While the HLA region is the most significant genetic risk factor, a multitude of other genes also contribute to T1D susceptibility.

After the HLA genes, the *INS* locus confers the highest genetic risk of developing T1D (**Figure 1**) (7). Studies have reported at-risk SNPs at the rs1004446 and rs7285903 loci within the *INS* gene and the 11p15.5 genetic region—harboring the *INS* and the *INS-IGF2* genes—respectively (56, 62). The strongest association is found within the *IDDM2* locus, which corresponds to the allelic variation at the *INS* variable number of tandem repeats (VNTR) (59). This variation is grouped into two main classes, the shorter class I and the longer class III. Homozygosity for class I increases T1D susceptibility by two- to five-fold, while class III is dominantly protective (63). Studies have found that this difference in susceptibility is caused by higher expression of the *INS* gene and proinsulin protein in the thymus and lower expression in the pancreas of human patients harboring the class III allele compared to class I expression. Therefore, higher proinsulin levels in the thymus are hypothesized to induce negative selection of insulin-specific thymocytes, thereby increasing tolerance to insulin autoantigens (63, 64). However, class III protection is not absolute, with an allele frequency of about 15% in T1D patients (65). Despite that, patients carrying this protective variant showed lower insulin autoimmunity, residual β cell function, higher C-peptide levels, better glycemic control, and a lower risk for diabetes complications (65, 66). These findings highlight the critical role of the *INS* locus in central tolerance and demonstrate that genetic variants can impact both disease risk and progression, underscoring the need to investigate other non-HLA genes associated with T1D.

Moreover, genome-wide association studies (GWAS) across different ethnic groups have identified polymorphisms in over 70 non-HLA loci, some of which include key protein-encoding genes that have been shown to play an immune-associated function (**Figure 1**) (56, 67, 68). For instance, the rs2476601 coding variant of *PTPN22* (R620W) alters T cell activation and proliferation. Its encoded protein, Lymphoid phosphatase (LYP), normally interacts with the T cell receptor (TCR)-modulating tyrosine kinase CSK, but this variant disrupts that interaction (69–71). However, there is no consensus on whether this SNP confers a gain- or loss-of-function, with contradicting findings on its effects on T cell activation (70–73). More recently, Anderson et al. clarified this dilemma by using CRISPR-Cas9 to introduce the risk variant, a non-risk control, and a *PTPN22* knockout into human cord blood-derived naïve T cells from the same donor. They concluded that the risk-edited cells increased a T cell activation profile following non-specific TCR engagement, which mimicked the observations exhibited by *PTPN22* knockout cells, suggesting the risk variant acts as a loss-of-function mutant that reduces the ability of LYP to inhibit TCR activation (74). More specifically, they reported increased TCR activation in self-reactive T cells with no significant changes in proliferation or cytokine production in T cells expressing high-avidity TCRs (74). The R620W *PTPN22* variant has also been shown to increase total Treg and naïve Treg subgroups in T1D patients compared to healthy controls, which is counterintuitive in the context of T1D (75). However, this variant has also been shown to decrease IL-2 signaling, which is essential for Treg suppressive function (76). Therefore, the increased Treg frequencies may be a compensatory response to dysfunctional Tregs, but this remains to be tested. Future studies should utilize *in vivo* models to compare the Treg population frequency when comparing the R620W variant to an appropriate control.

Two T1D-associated at-risk variants, rs11203203 and rs80054410, in another regulator of T cell activation and LYP interacting-molecule, *UBASH3A*, have been shown to increase mRNA expression in human primary CD4+ T cells, thereby reducing IL-2 production by inhibiting NF-κB signaling (77, 78). Another variant, rs11203202, has also been associated with T1D risk, although its specific mechanism remains unknown (79). On the other hand, rs1893592, a protective *UBASH3A* variant, has been reported to reduce *UBASH3A* gene expression in human primary CD4+ T cells, which in contrast increased IL-2 secretion (79). However, complete *UBASH3A* deficiency accelerates T1D onset in NOD mice and rats (80, 81). Together, these findings demonstrate that variants in genes like *PTPN22* and *UBASH3A* can disrupt critical immune checkpoints, leading to the hyperactivation of T cells and a breakdown of regulatory mechanisms essential for preventing autoimmunity.

*SIRPG* levels may also be relevant to the onset of T1D. *SIRPG* encodes SIRPγ, a member of the signal regulatory protein (SIRP) family (82). Two SNPs within this gene have been associated with T1D risk: the rs6043409 loci and the intronic rs2281808 (82). *SIRPG* produces multiple transcript isoforms via alternative splicing, with isoform 1 being the most expressed in CD4+ and CD8+ T cells (83). The major ‘G’ allele at rs6043409, which confers risk for T1D, was found to be associated with higher cell-surface levels of isoform 1 SIRPγ on both CD4+ and CD8+ T cells, while the minor ‘A’ allele reduced SIRPγ expression in a dose-dependent manner (83). This study also found that *SIRPG* knockout in Jurkat T cells resulted in fewer T cell conjugates, which suggests a role in T cell to T cell interactions (83). However, another group found that the presence of the T1D-linked genetic variant of rs2281808 is associated with reduced SIRPγ expression in CD8+ T cells, an increased effector state with a lower T cell activation threshold, and a reduction in gene expression associated with long-term functional memory T cell formation (84). Further studies must investigate this observation, considering the various isoforms of SIRPγ, and the long-term maintenance of this effector T cell population *in vivo* with a reduced memory T cell pool.

Patients with autoimmune diseases, including rheumatoid arthritis, systemic lupus erythematosus, multiple sclerosis, and T1D, have been shown to have lower levels of *CTLA4* mRNA (85). The encoded molecule supports Treg differentiation, reduces effector T cell function, enhances the ability of Tregs to reduce immune responses, increases anti-inflammatory cytokine secretion, and reduces pro-inflammatory cytokine secretion (86–88). The T1D-associated rs231775 variant was associated with a younger age of onset and higher prevalence of ketoacidosis at clinical diagnosis (89). It has also been shown to inhibit CTLA4 trafficking to the cell surface, which may result in lower protein expression in naïve T cells and Tregs (90, 91). As a result, Treg function may be negatively impacted, allowing for increased effector T cell function and subsequent secretion of the pro-inflammatory cytokines tumor necrosis factor (TNF) α and IFNγ (89).

Another critical regulator of various immunological processes associated with multiple autoimmune disorders is CD226, a member of the immunoglobulin superfamily (92). It competes with the inhibitory receptor T cell immunoreceptor with Ig and ITIM domains (TIGIT) for CD155 and CD112 binding on APCs. Upon binding, CD226 acts through VAV1 to induce ERK signaling critical in augmenting TCR activation and perhaps natural killer cell activation (7, 92, 93). CD226 activation also leads to increased release of various cytokines by CD4+ T cells, including IL-5, IL-10, IL-17, IFNγ, and IL-13 (93). The T1D-associated SNP at rs763361 increases VAV1 phosphorylation and subsequent IL-17 signaling, which is involved in T1D pathogenesis, in CD4+ T cells, suggesting an increased functional variant (93). A global knockout in female NOD mice reduced islet infiltration in pre-diabetic mice, as well as FOXP3-deficient Tregs and the incidence of T1D (94, 95). In a Treg *Cd226* conditional knockout model, female NOD mice also displayed decreased insulitis and diabetes incidence, supporting the notion that CD226 is involved in Treg stability (95). Treatment with an anti-CD226 monoclonal antibody reduced proliferation of CD4+ T cells and of CD8+ effector memory T cells in the spleen of female NOD mice, as well as the frequencies of autoreactive CD8+ T cells in the pancreas (96). This was partly due to enhanced Treg suppressive function of CD4+ responder T cells. A recent study found that TIGIT+CCR7- Tregs, which correlated positively with β cell function, secrete high levels of TGF-β1, acting as a potent immunosuppressive signaling specifically targeting and inhibiting the functional activity of cytotoxic CD226+CCR7- CD8+ T cells in mice (97). Future efforts should focus on using anti-CD226 monoclonal antibodies to delay T1D onset in AAb+ nondiabetic patients.

### 3.2 Genetic influence on environmental response: the *IFIH1*, *TYK2*, and *FUT2* genes

Among the genes linking genetics to environmental triggers, Interferon-Induced with Helicase Domain 1 (*IFIH1*) stands out for its direct role in the immune response to viruses, a major suspected trigger for T1D. *IFIH1* encodes melanoma differentiation-associated protein 5 (MDA5), a ubiquitously expressed cytoplasmic RNA helicase and sensor that is crucial to survival after systemic viral infection by inducing an MAVS-mediated type I IFN immune response (98). It detects double-stranded RNA (dsRNA) generated by viruses from several families, such as Picornaviridae, Coronaviridae, Flaviviridae, and Paramyxoviridae (99). MDA5 is a member of the RIG-I-like receptors family that detects and forms filaments along the length of long dsRNA (0.5–7 kilobases) to activate the innate immune response (100–102). It contains a DExD/H-box RNA helicase domain and a C-terminal domain (CTD) that bind dsRNA, along with two N-terminal caspase activation and recruitment domains (CARDs) that interact with the mitochondrial antiviral signaling protein (MAVS) to induce transcription of type I IFNs and downstream IFN-stimulated genes (ISGs) (99, 103). When MDA5 detects viral dsRNA, the CARD domains undergo ISG15-mediated ISGylation, facilitating MDA5 oligomerization onto the RNA and subsequent CARD clustering and binding to MAVS (99, 104).

GWAS studies have linked at least four SNPs within *IFIH1* to T1D risk and protection (105). In peripheral blood mononuclear cells (PBMCs), E627* (rs35744605), A843H (rs3747517), and I923V (rs35667974) were associated with protection against T1D by reducing type I IFN-associated gene transcription following stimulation with IFNβ or poly(I:C), a MDA5 agonist (106, 107). The E627* change results in a premature stop codon at the second helicase of the MDA5 protein. Therefore, the mutant lacks part of the second helicase domain and the entire CTD domain (108). It reduces *IFIH1* gene expression, resulting in a complete loss of dsRNA binding activity (106, 108). A study investigating the effects of *Ifih1* deficiency used NOD mice with an *Ifih1* helicase 1 domain deletion or a premature stop codon, resulting in a functional gene knockout. They showed that T1D developed similarly in female wild type and knockout mice, with a significant delay in T1D onset and reduced insulitis in helicase 1 deleted male and female mice (109). When infected with coxsackievirus B3 (CVB3), wild type and knockout female and male mice had accelerated T1D onset, while helicase 1 deleted mice exhibited delayed onset (109). In another study, NOD mice heterozygous for an *IFIH1* knockout allele were completely protected from T1D development following CVB4 infection. Viral clearance was similar between wild type and heterozygous groups, but the latter had a unique type I IFN signature with an early burst of IFNβ followed by a decrease, different from the steady rise in wild type mice (110). This signature appeared to shift the balance of T cell responses away from an effector response and toward a Treg response, which helped suppress IFNγ-producing CD4+ T cells (110). Like these genetic models, the I923V variant may also be protective through reduced MDA5 function. The SNP was shown to have minimal conformational or stereochemical effects on MDA5 itself but is thought to impair the CTD domain within MDA5, weakening the interaction of the protein with dsRNA. This weakened activity may impact filament assembly and subsequent MAVS activation, thereby inducing a lower IFN signal upon activation (106, 107, 111). However, other studies have shown that the I923V variant does not affect dsRNA binding activity but still exhibits reduced biological activity, suggesting an alternative signaling process (108). Regardless, these data suggest that protective variants may function partly through lower expression and/or function of MDA5.

The rs1990760 locus has been implicated to harbor a T1D risk allele that results in an amino acid change from alanine to threonine at codon 946 (A946T) within the CTD of *IFIH1* (112). A study found A946T and H843R (rs3747517) together increased IFNβ production in PBMCs stimulated with poly(I:C) (113). This haplotype also increased basal type I IFN signatures in mice with no differences in *Ifih1* gene expression and enhanced resistance and survival against encephalomyocarditis virus (113, 114). In both male and female NOD mice homozygous for the risk haplotype, T cell populations were altered. Although diabetes incidence showed only a modest increase in all mice, the study found a significant acceleration of T1D onset in females and an increase in insulitis in males (114). The risk haplotype also manifests an embryonic survival defect, a common feature in individuals with an enhanced response to endogenous dsRNAs (113). More recently, the A946T variant alone was confirmed to lead to increased basal type I IFN activity in reporter cell lines due to increased MDA5 ATPase activity, which may enhance filament formation on dsRNA and downstream signaling (115). These findings suggest that the *IFIH1* A946T variant, with or without the presence of the H843R variant, contributes to T1D progression and sex-specific immune responses by increasing MDA5 activity.

The evidence from both protective and at-risk *IFIH1* variants highlights the critical role of the type I IFN pathway in T1D pathogenesis. Interestingly, transient type I IFN gene expression in peripheral blood is a suggested risk factor preceding autoantibody seroconversion and autoimmunity in children with a genetic predisposition to T1D, though not all children progress to the disease (116, 117). Type I IFN signaling has also been shown to be significantly elevated in T1D ductal cells when compared to control and AAb+ ductal cells (20). This signaling is implicated as a driver of HLA class I hyperexpression in β cells via IFN-stimulated regulatory elements found in the proximal promoter of *HLA-A*, *HLA-B*, and *HLA-C* (42, 118). A study using EndoC-βH1 cells found that IFNα treatment leads to inflammation, ER stress, and an increased expression of HLA class I proteins. While IFNα alone did not induce cell death in these cells, indicating that IFNs alone are not a sufficient trigger, it did increase apoptosis when combined with IL-1β (119). Blockage of IFNα signaling in NOD mice enhances CD4+ T cell-mediated production of IL-4 and IL-10 but not IFNγ, which helps suppress T1D (120). This signaling cascade, initiated by MDA5 and other sensors during viral infections, culminates in the activation of the JAK-STAT pathway, which is critically dependent on the non-receptor tyrosine kinase 2 (TYK2).

Genetic polymorphisms in *TYK2* that reduce its function are associated with reduced risk of T1D and other autoimmune diseases (7, 56). The protective variant at rs2304256 was shown to decrease STAT1 phosphorylation in B lymphoblastoid cell lines when stimulated with IFNα (67). In this same study, *TYK2* knockdown in EndoC-BH1 and primary human islet cells decreased apoptosis, STAT1 and STAT2 phosphorylation, IFNα and CXCL10 secretion, and class I HLA protein expression (67). The rs12720356 (I684S) and rs3456443 (P1104A) have also been implicated in T1D and other autoimmune diseases, including psoriasis, rheumatoid arthritis, and Crohn’s disease (121). However, the minor allele at rs12720356 was found to have no effect on STAT phosphorylation or cytokine production, while the protective allele at rs3456443 did reduce phosphorylation and cytokine stimulation in peripheral blood immune cells (121). *Tyk2* knockout in NOD mice impaired the expression of T-BET, a transcription factor vital for cytotoxic T lymphocyte (CTL) development, in isolated CD8+ T cells stimulated with IL-12, as well as the cross-priming of autoreactive CTLs by resident dendritic cells in the pancreatic lymph node. As a result, *Tyk2* knockout mice exhibited reduced proliferation of autoreactive CD8+ T cells and impaired cytotoxic activity against β cells (122). TYK2 inhibition in human β cells prevented IFNα-induced ER stress, cytokine-mediated apoptosis, and upregulation of HLA class I molecules and CXCL10, thereby limiting pathological interactions between β cells and T cells (123). In NOD mice, it delayed T1D onset, reduced insulitis, inhibited the expansion of T-BET+ CTLs, and promoted a T cell exhausted signature in the spleen and pancreatic lymph nodes (122, 123). However, a significant reduction of TYK2 function makes patients more prone to infections and makes virus-resistant mouse models sensitive to virus-induced diabetes (121, 124). While genes like *IFIH1* and *TYK2* modulate the response of the immune system to pathogens and inflammatory signals, other genetic factors shape the direct interaction between the host and the environment. This is exemplified by the *FUT2* gene, which influences the composition of the gut microbiome.

The *FUT2* gene encodes an enzyme responsible for creating the type 1 H antigens. These antigens are the precursors for the ABO and Lewis b histo-blood group antigens, which are found on the surface of intestinal and other epithelial cells (125). A common loss-of-function variant at rs601338 (W143*), results in a non-secretor phenotype in homozygotes, meaning these individuals cannot secrete ABO antigens in their gut lumen and other secretions (126). This non-secretor genotype is a well-established risk factor for T1D and is associated with a different gut microbial community compared to secretor individuals (127, 128). The altered microbiome in non-secretors can increase the susceptibility of the host to bacterial, fungal, or viral infections, as well as kidney disease and chronic inflammatory diseases (126, 129). This heightened inflammatory state within the gut, caused by environmental and systemic triggers, could potentially contribute to a breakdown of immune tolerance. In fact, T1D has been linked to alterations in the gut microbiome, including lower microbial diversity (130). These changes can impact both mucosal integrity and immune tolerance, leading to greater gut permeability, which has been reported to precede clinical onset of T1D (131). This increased permeability, caused by molecular mimicry and a modulated gut immune system, is thought to increase the risk of T1D by potentially inducing an autoimmune response (57). Considering this, the *FUT2* gene represents a critical genetic link between the gut microbiome and T1D pathogenesis. Despite that, the role of these variants within *IFIH1*, *TYK2*, and *FUT2* in islet health and biology remains unknown. To further elucidate the role of these genes in T1D pathology, future studies should utilize islets from patients harboring these variants or genetically engineered stem cell-derived islets and treat them with various stressors to delineate unique responses.

The genetic landscape of T1D is complex, dominated by HLA and *INS* loci that govern immune tolerance, but also involving non-HLA genes that modulate the host’s response to environmental factors like viruses and the gut microbiome. This interplay highlights the necessity of exploring specific potential environmental triggers in detail.

## 4 Enterovirus infection as an environmental trigger of T1D

There have been reports associating T1D with lower microbial diversity, diet, low vitamin D exposure, and exposure to toxins and chemical compounds in food or water (130). Moreover, T1D follows a seasonal variation pattern where new diagnoses in children peak in autumn and winter months and trough in summer months in both the northern and southern hemispheres (9, 132, 133). Considering this, viral infections, particularly by enteroviruses, have also been implicated as a potential environmental trigger for T1D (**Figures 1**, **2**) (6).

Enterovirus infection has been associated with T1D for decades (6, 134). Enteroviruses are members of the *Picornaviridae* family, which includes coxsackieviruses, polioviruses, and encephalomyocarditis virus. They are non-enveloped, single-stranded RNA viruses with small 7.5 kilobase genomes encoding 11 proteins (135). Coxsackieviruses are organized into 23 group A viruses and six group B viruses (CVB). Like T1D, coxsackievirus infections are mostly diagnosed in children. In mice, group A viruses are tropic for striated muscle, whereas CVB strains have tropism for the pancreas, heart, liver, brown fat, central nervous system, and striated muscle (136). Moreover, CVB is known for its persistent, chronic inflammatory infections, which can lead to pancreatitis, myocarditis, and meningitis in both humans and mice (137–139). Although the mechanisms governing how CVB infection triggers T1D remain unknown, infection induces a plethora of cellular signatures related to β cell stress and T1D, suggesting a potential link.

### 4.1 Enteroviral mechanisms: viral entry, replication, host response, and links to T1D

To understand how viral infections may potentially contribute to T1D, exploration of the fundamental mechanisms by which enteroviruses, like CVB, interact with host cells is essential. CVB has been reported to infect multiple human cell types associated with T1D, including primary islets, human pluripotent stem cell-derived islets, ductal cells, thymic epithelial cells, thymic fetal cells, and human PBMCs (115, 140–149). Understanding these interactions—from viral entry and replication to the subsequent host immune response—is crucial as they can drive cellular changes linked to T1D development.

Enteroviruses must attach to and enter a host cell to initiate viral replication. For some enteroviruses, including CVB3, decay-accelerating factor (DAF or CD55) serves as an attachment receptor that facilitates virus binding to the uncoating receptor, coxsackievirus-adenovirus receptor (CAR) (150–154). This receptor interaction with CAR induces conformational changes in the virion that destabilize the capsid, facilitating the release of the viral genome, which is then translated and replicated to generate new virions (153, 155).

CVB has evolved sophisticated strategies to manipulate and subvert host immune responses to ensure its replication and survival. The initial detection of viral dsRNA by MDA5 triggers expression of type I IFN-associated genes via MAVS, preparing the host for an antiviral response and limiting early viral replication (98, 156). However, the CVB3-encoded protease, 3C^pro^, has been shown to attenuate type I IFN signaling by cleaving MAVS and TRIF, another component of the antiviral response, within hours of infection in multiple cell lines (157). MAVS, alongside MDA5, are crucial to survival after systemic CVB infection. In C57BL/6 mice, the absence of either protein leads to deficient type I IFN production and early mortality (98). Furthermore, CVB non-structural proteins impair the Golgi Apparatus (Golgi) and increase the rate of host endocytosis in HeLa and ECN90 β cells. As a result, surface expression of B2M and class I HLAs is reduced, helping the virus evade identification and clearance of infected cells by cytotoxic CD8+ T cells (138, 158, 159). This multifaceted suppression of the host antiviral response illustrates how enteroviruses have evolved sophisticated strategies to persist and spread infection.

The mechanisms by which enteroviruses spread from cell to cell and disseminate within a host are critical to understanding how they cause disease. Studies have shown that cell lysis is not the only method enteroviruses use to spread infection to other nearby cells. CVB viral proteins have been shown to colocalize with extracellular microvesicles, phosphatidylserine vesicles, exosomes, mitophagosomes, and autophagosomes, indicating the virus actively directs itself to these cellular compartments (160–164). These virus-containing structures are then ejected from the host cell, effectively spreading infection in a non-lytic manner to both entry receptor-positive and -negative cells. This mechanism allows for the simultaneous transmission of multiple viral particles to a single new host cell, thereby increasing infectivity (163). For extracellular microvesicles, mitophagosomes, and autophagosomes, this release mechanism is dependent on the autophagy pathway (160, 162, 165).

CVB infection has been observed to induce autophagosome formation while blocking autophagosome-lysosome fusion in both *in vitro* and *in vivo* models (160, 161, 166). This process facilitates viral replication within an enclosed membrane, allowing the virus to evade cytoplasmic RNA sensors and the subsequent immune response. CVB3 also uses its protease 2A^pro^ to cleave the SQSTM1 host protein, which is essential for selective autophagy and host defense. While this cleavage impairs the ability of cells to degrade misfolded proteins, the resulting fragments also compromise host defense by losing the ability to activate the NFκB pathway (167). Together, these findings demonstrate how enteroviruses leverage host cellular machinery, not just for replication, but for their own dissemination and immune evasion, providing a compelling picture of how these viruses, specifically CVB, may contribute to the chronic inflammation and β cell destruction characteristic of T1D.

### 4.2 Evidence of persistent enterovirus infection in T1D patients

The hypothesis that a persistent enteroviral infection may be a key driver of T1D onset has gained significant traction. While a direct mechanistic link has yet to be found, CVB infections are known to cause chronic inflammatory diseases, including myocarditis, meningitis, and pancreatitis (137). Evidence from a case-control study in Finland, which analyzed samples from the Type 1 Diabetes Prediction and Prevention study, reported enteroviral RNA in the stool of children up to one year before they developed islet autoimmunity (11). Similarly, the Diabetes Virus Detection Study (DiViD) examined pancreatic tissue from six live, recent-onset T1D patients and found a low-grade enteroviral infection in the pancreatic islets of all six samples (**Figure 2**) (48). Moreover, a recent phase 2, placebo-controlled, randomized clinical trial tested whether antiviral therapy could preserve β cell function in children and adolescents with recent-onset T1D. They found that six months of treatment with pleconaril and ribavirin helped preserve residual insulin production in treated patients compared to those in placebo groups (168). Together, these findings suggest that an initial acute enteroviral infection fails to be fully cleared, instead transitioning into a low-grade, chronic state within the pancreas.

The DiViD has confirmed enterovirus presence in their T1D cohort at a higher degree than in nondiabetic controls in PBMCs, duodenal biopsy, and stool (169). They set out to determine if other viruses could be detected by measuring the relative expression of viral genomes from more than 20 human viruses other than enteroviruses. Only one patient tested positive for another virus, Epstein-Barr virus (170). Their study also showed that 1% of pancreatic cells expressed viral antigens (**Figure 2**). More importantly, they confirmed the transmission of enterovirus from pancreatic tissues to uninfected cells, suggesting the presence of infectious virus in the pancreas of recent-onset T1D patients (170). Gene expression analysis of islets of this newly diagnosed T1D cohort showed significant enrichment of pathways associated with viral reproduction, infectious cycles, and cellular stress (171). Independent studies also found that several ISGs were overexpressed in islets from T1D and AAb+ patients compared to controls, following an expression pattern similar to that of islets exposed to CVB (149, 172, 173). Other studies have confirmed a significant increase in the number of enteroviral RNA-containing cells in the pancreas of T1D and AAb+ individuals compared to control groups but reported that most virus-positive cells were found within the exocrine pancreas (148, 174). Recently, our group has demonstrated that CVB can infect all cells found in primary human islets with ductal cells expressing the strongest type I IFN-associated transcriptional signature (149). These results highlight the interplay between different cell types and the potential role of nonendocrine cells in contributing to T1D progression.

Future research is needed to validate the initial findings suggesting a link between persistent enteroviral infection and T1D onset by conducting larger, multicenter studies to increase statistical power and overcome the limitations imposed by small sample sizes in current pancreatic tissue and interventional trials, including the DiViD. A critical next step involves longitudinal studies to definitively determine if the presence of enterovirus in the pancreas precedes or is a consequence of T1D-associated autoimmunity. Furthermore, researchers should focus on identifying and characterizing the cellular viral reservoir within the pancreas, particularly the role of non-endocrine cells, to develop cell-specific antiviral strategies that prevent viral persistence and interrupt the chronic inflammatory cycle.

### 4.3 Viral susceptibility: genetic and cellular factors in T1D patients

The microbiome and genetic background of T1D patients may also make them more susceptible to viral infections. Before system spread, the primary replication site of CVB is the mucosa found in the gastrointestinal or the upper respiratory tracts (6). The altered microbiome reported in T1D patients before clinical onset impacts mucosal integrity, leading to gut permeability (131). Thus, the gut of infected T1D susceptible patients may serve as a viral reservoir that could promote chronic inflammation. In a study of pancreatic tissue, CAR surface receptor expression was higher in the islets of T1D and AAb+ patients compared to controls. The research also revealed a possible feedback loop in which viral-mediated or T1D-associated inflammation increases CAR expression, making the islets more vulnerable to further viral spread (175). This increased CAR expression may be in part due to the location of a predominant isoform, CAR-SIV, within the insulin secretory granules in the cytoplasm of human β cells (176). Confocal and electron microscopy showed that CAR-SIV colocalized with insulin, ZnT8, and PC1/3 within insulin granules. Therefore, insulin granules may serve as additional viral replication hubs utilized for viral spread in β cells (176). T1D patients have also been found to have a marked reduction or absence of several central defensins in their pancreas, regardless of the level of inflammation, whereas controls had increasing defensin expression with increasing inflammation (177). Defensins are potent antimicrobial peptides that play a role in both innate and adaptive immune systems. Therefore, the reduced expression of defensins could lead to prolonged viral-mediated inflammation and dysregulation of the immune system, potentially contributing to the development of T1D. Additionally, PBMCs from individuals carrying the *IFIH1* 946T risk allele were more likely to test positive for enteroviral RNA than those with the protective allele (148). Together, these findings show that T1D patients may be susceptible to persistent viral infections due to factors that favor chronic infection.

Future studies should investigate the functional consequences of increased CAR-SIV expression in human β cells, specifically examining its role in promoting viral persistence and spread within the insulin secretory granules. Research is also needed to directly establish a link between T1D-associated changes in the gut microbiome and the enhanced susceptibility to or clearance of enteroviral infection in the enteric mucosa, which may serve as a reservoir for system viral spread. Furthermore, mechanistic studies should elucidate how the T1D-associated reduction in pancreatic defensins impact viral load, local immune response kinetics, and the ultimate extend of β cell damage following CVB infection.

### 4.4 Molecular mimicry, central tolerance, and β cell stress: plausible mechanisms for the association of enteroviral infections with T1D

Enteroviral infection may induce T1D through multiple mechanisms that have yet to be validated in T1D models. The host’s genetic predisposition, particularly with genes that govern immune tolerance, may impact the immune response to viral infection. The combined inflammatory impact of cytotoxicity by CD8+ T cells, immune-mediated perturbations of the islet ECM, and other mechanistic changes lead to β cell death and decreased β cell mass in a time-dependent manner (**Figure 2**). This creates increased metabolic demand on remaining β cells that try to compensate for the reduced β cell mass by increasing insulin transcription and output (35, 178). As cell mass continues to decrease, the increased metabolic demand, continuous exposure to pro-inflammatory cytokines, and sustained hyperglycemic conditions upregulate mitochondrial and ER stress in β cells, which further impair β cell function and promote stress-mediated stress (179–188). Multiple CVB variants have been shown to induce cell degeneration, pyknosis, reduction of islet identity genes, and decreased insulin content and secretion in primary human islets cultured *in vitro* or transplanted into diabetic NOD mice (141, 143). Considering this, CVB infection, particularly persistent infection, may contribute to the overall demise of the β cell population by further activating autoreactive T cells, augmenting the effects of autoimmunity, and by further inducing β cell stress and death.

CVB-induced β cell death may be partly caused by virus-induced ER stress since ER stress negatively impacts β cell function and viability, leading to T1D onset (184, 188). Increased virus-induced Golgi and ER stress may also contribute to autoantigen production through improper protein processing. However, these observations have not been reported in human primary islets. Future mechanistic studies should use CVB-infected human islets to quantify the production, processing, and display of key autoantigens, utilizing proteomics or other methods, following infection-induced ER stress and comparing results to an inactivated virus as a control.

CVB-induced immune responses may also contribute to islet autoimmunity. CVB5-infected primary human islets exhibited differential expression of 33 microRNAs predicted to target 57 candidate T1D risk genes involved in T cell activation and maturation, as well as immune response (145). Yet, the mechanisms of action of microRNAs and other non-coding RNA in specific CVB-infected islet cell types remain unknown. To address this gap, microRNA gain and loss-of-function studies could be performed in specific islet cell populations using immortalized cell lines or whole islets or with human pluripotent stem cells-derived islets from T1D patients to account for genetic susceptibility. These experiments would determine how microRNAs modulate T1D-associated gene expression and viral replication kinetics, which could elucidate a mechanistic link between enteroviruses and T1D. Given that β cells also express about 80% of T1D risk genes, they should be the specific focus of study in this context alongside CD8+ and CD4+ T cells (67, 189, 190). Addressing these mechanistic questions will help determine if persistent CVB infection leads to the immunological changes associated with the innate and adaptive immune systems of T1D patients through the upregulation of multiple T1D-associated genes.

Viral peptides from CVB have also been shown to harbor molecular mimicry with T1D autoantigens. CVB4 P2-C protein has sequence homologies to GAD, a T1D autoantigen, and is recognized by TCR receptors on T cells against GAD65 (**Figure 2**) (191, 192). Although CVB downregulates class I HLAs, β cells still present HLA bound viral peptides that mostly map to the P2-C protein and are conserved across CVB1 and CVB3 (159). Moreover, some of these viral peptides are recognized by CD8+ T cells, with a fraction of these cells also recognizing a homologous GAD sequence (159). However, this cross-reactivity is not indicative of CVB-mediated T1D induction. Although, CVB infection may prime T cells to cross-react against a protein sequence that mimics a self-antigen, AAb are not causative and have mostly been used as biomarkers (14, 15, 19). Thus, molecular mimicry as a pathological mechanism is unlikely.

Moreover, CVB was shown to persistently infect a human pancreatic ductal cell line (147). Persistent CVB1 infection of PANC-1 cells upregulated type I IFN and immune response genes and induced differential expression of genes associated with the ECM, lysosomal biogenesis, β cell-to-cell communication, and hormone secretion (147). We have also shown that ductal cells found within isolated primary human islets exhibit a similar transcriptional signature as PANC-1 cells, particularly a strong transcriptional signature associated with type I IFNs, which may contribute to the cytokine storm preceding disease onset (116, 149). In addition, multiple studies have shown the integrity of the ECM and islet microenvironment is connected to the T1D pathogenesis by promoting β cell survival and blocking T cell infiltration into islet tissue (43). However, interactions between the multiple pancreatic cell types and the changes in the islet microenvironment in a human CVB infection model have not been investigated. Future work using co-culture experiments is needed to study the effects of the exocrine compartments on the pancreatic endocrine cells, providing insight into the impact that the pancreatic microenvironment has on islets following viral infections. Specifically, researchers should use advanced imaging and proteomics on infected co-culture models to characterize the specific changes in ECM composition and structure following ductal cell infection. Additionally, the impact of the ductal IFN signature on β cell function needs to be assessed. This could be done by measuring β cell insulin secretion, content, and viability when exposed to conditioned media from infected ductal cells, thereby isolating the effects of soluble mediators.

CVB-associated T1D may also be caused by the viral-associated effects on central tolerance. CVB4 was reported to persistently infect primary cultures of thymic epithelial and fetal cells, leading to higher inflammatory cytokine and class I HLA expression and decreased *IGF2* gene and protein expression (25). *IGF2* expression in the thymus is important because it plays a role in insulin tolerance by serving as a self-antigen of the insulin family during T cell maturation and selection against autoreactive T cells (25). Thus, CVB4 infection of the thymus could lead to decreased selection against autoreactive T cells, which increases the risk of developing islet autoimmunity. Future research should utilize humanized mouse models, infect them with CVB, and track the maturation of T cells to determine if matured T cells show an increased reactivity toward β cell antigens compared to T cells form uninfected controls.

### 4.5 Studies supporting no association between enteroviral infection and T1D

A major limitation in the field is that the majority of data supporting an association between enteroviral infection and T1D have been obtained from epidemiological studies, and a definitive mechanistic link remains to be fully elucidated. To provide a balanced view, various studies have found no association between enteroviral infections and either islet autoimmunity or recent onset T1D. These null findings have been reported using different sample types, including fecal samples used to investigate the intestinal virome, as well as intestinal biopsies, plasma, and serum (193–198). However, a key limitation of most of these studies that reported no association is their small sample sizes and the lack of longitudinal sampling necessary to capture transient viral infections. In contrast, a recent and comprehensive meta-analysis reviewing 60 studies, which included 5981 individuals with either islet autoimmunity or T1D, did find a significant association between enteroviruses and cases of AAb+, established T1D, and recent onset T1D (199). This suggests that while individual smaller studies may fail to detect the link, the cumulative evidence supports an association. Overall, substantial epidemiological and tissue evidence supports the potential involvement of enteroviral infection in T1D. However, new models must be developed to elucidate the sophisticated viral mechanisms that converge with β cell stress and genetic risk to definitively provide a link between enteroviruses and T1D initiation.

## 5 Disease modeling using human pluripotent stem cells

Although most of the available data for our understanding of T1D have been generated using animal models, primary islets, and immortalized cell lines, there are limitations to these models. Even though most aspects of the pancreatic transcriptome are conserved between mice and humans, there are key differences in the gene expression patterns of transcription factors and islet architecture. Since isolated primary human islets cannot be cultured for extensive periods of time and exhibit donor-to-donor variability, most groups have historically relied on rodent models and cell lines to robustly understand the genetic and mechanistic components involved in the initiation and progression of T1D. However, there are concerns regarding how applicable rodent data are to human islets. Single-cell RNA sequencing data have shown that some genes expressed in mouse islets are differentially expressed or completely undetected in human islets and vice versa (200). Islet architecture and composition are also very different between these species. In rodent islets, β cells are in the center, surrounded by the other endocrine cells, and make up about 60-80% of the islet. Whereas in human islets, there is a significantly higher proportion of *α* cells as well as a random distribution of endocrine cells (201). Considering this, modeling enteroviral infections in NOD mice and other murine models does not capture the whole impact of viruses in a human context. Thus, additional models are necessary to validate findings from rodent models in humans.

Human pluripotent stem cells (hPSCs) have proven very valuable in the study of diabetes (202). Many groups in the field have developed multiple step-wise protocols using small compounds and proteins to recapitulate pancreatic development and differentiate hPSCs into SC-islets (203–212). These SC-islets are often more than 80% endocrine, capable of secreting insulin following glucose stimulation, and capable of restoring normoglycemia after transplantation into diabetic mice (203, 204, 207). Furthermore, single-cell sequencing has been successfully used extensively to understand the biology of these tissues (209, 213–217). SC-islets can be generated in abundance, have the same genetic background across batches, and provide a human-specific model. Additionally, comparative analyses of directed differentiation protocols derived from multiple groups have shown that they generate transcriptionally similar SC-islets (218). These characteristics allowed the field to conduct replicable and robust experiments, such as generating SC-islets from patients with T1D and subjecting them to environmental stressors (219, 220).

SC-islets provide us with the opportunity to study cells that are otherwise destroyed and unavailable in T1D patients. For instance, patients with Wolfram Syndrome (WS) have rare autosomal recessive mutations in their *WFS1* gene that give rise to multiple complications, such as juvenile-onset diabetes, optic atrophy, and deafness (185). *WFS1* encodes an ER resident membrane protein that normally functions to attenuate the ER stress response in β cells but increases ER stress and apoptosis when mutated in these cells (185, 221). WS-derived diabetes is not typically studied using WS β cells because of cell death during disease progression and the rarity of the disease. However, CRISPR-Cas9 was used to correct pathogenic variants within the *WFS1* gene of hPSCs originally from several WS patients. Unlike the unedited cells, the genetically corrected cells generated SC-islets with improved differentiation efficiency at the endocrine cell induction stage, reduced ER and mitochondrial stress, and increased function (222). These results led to an improved understanding of WS and identification of a drug combination to help patients, which is currently in an ongoing phase 2 clinical trial (223, 224). These findings suggest that patients develop diabetes partly by generating fewer functional β cells due to cell stress and developmental issues.

SC-islets also allow for genetic modeling of candidate genes, SNPs, and mutations (202, 225). As mentioned above, we have used SC-islets to study the effects of a genetic candidate, *MIR7-3HG*, following CVB3 infection (149). Our group has modeled the effects of cytokine, ER, and Golgi stress on the various cell types found within SC-islets (226). Another recent study has utilized single-cell sequencing of CVB4 and cytokine-stressed primary human islets, identifying associations with T1D-associated GWAS signals in *DLK1*/*MEG3*, *RASGRP1*, and *TOX*, and used SC-islets to validate the mechanistic relevance (227). Others have used a similar approach to understand how β cell development and health are affected by insulin mutations or SNPs derived from monogenic patients, neonatal diabetes patients, and GWAS studies (228–238). SC-islets from T1D patients have been generated to study the effects of IFN-*γ*, TFN-*α*, and IL-1β, which are pro-inflammatory cytokines involved in autoimmune-mediated β cell injury in T1D (46, 219). Together, these studies show the power of hPSCs and SC-islets to model various disease contexts. These advanced *in vitro* systems are crucial for validating the complex interplay between genetic risk, viral mechanisms, and β cell pathology identified in epidemiological studies, moving beyond correlational data to establish a causal mechanism.

## 6 Future directions and conclusions

The major takeaways of this review are:

- T1D results from a breakdown of central and peripheral immune tolerance, enabling autoreactive T cells to activate, infiltrate pancreatic islets, and destroy β cells.
•Genetic susceptibility shapes how the immune system responds to pathogens and inflammatory cues.
•Enterovirus infection, particularly by CVB, is a strong candidate environmental trigger, with evidence that viral persistence, β cell stress, and immune evasion mechanisms may accelerate or initiate autoimmune responses.
•β cells play an active role in disease progression, as they express many T1D-associated genes, upregulate stress and inflammatory programs, and increase immune visibility under inflammatory or viral stress.
•Advanced human model systems, especially SC-islets, are essential for establishing causal mechanisms, enabling researchers to link genetic risk, viral stressors, and immune responses in a human-relevant context and guide the development of targeted preventive therapies.

At the time of clinical onset, T1D patients have lost nearly 80% or more of their insulin-secreting β cells to inflammation, immune-mediated ECM degradation, β cell stress, and other mechanisms (**Figure 2**) (239). Immune-based pharmacological therapies have been the most effective at delaying T1D progression and preserving β cell mass and function but are unable to prevent disease pathology completely (21–23, 45). To develop a preventive cure, a deeper understanding of the mechanisms behind environmental triggers, autoimmunity, central and peripheral tolerance, as well as immune-mediated β cell death is necessary.

To address these limitations and advance toward a preventive cure, future research must focus on several key knowledge gaps in immune-mediated T1D pathogenesis in a human context. The field has limited understanding of how heterogeneity and differences in genetic susceptibility across different ethnic groups and countries contribute to T1D (240–242). Expanding this knowledge will lead to improved genetic risk scores and personalized therapies as new relevant loci are discovered, providing a better picture of the genes and regulatory elements involved in T1D onset and progression. This effort is particularly important since at-risk genes have been associated with the order and age at which certain AAb appear. For instance, The Environmental Determinants of Diabetes in the Young (TEDDY) study has identified that GAD AAb arise first in children with HLA-DR3/3 haplotypes, while insulin-only AAb were more prevalent in HLA-DR4/8 patients (14, 15, 243). The study has also determined that rs2476601 within *PTPN22* was the best predictor for insulin AAb appearing first and that rs231775 at *CTLA4* is correlated with GAD AAb at initial seroconversion (14, 15, 243). However, these datasets are largely composed of patients from European ancestry, highlighting the immense value of including more diverse populations to achieve a global understanding of T1D and ensure that current models and therapies are applicable and effective for all patients. This expanded understanding of gene-AAb relationships and ethnic differences is essential for advancing personalized medicine in T1D. Integrating detailed genetic risk profiles with individual AAb signatures could allow for precision risk stratification, identifying individuals most likely to progress rapidly. Ultimately, this approach would support the development of tailored, pre-emptive therapies, such as specific antiviral drugs or immune modulators, administered only to those patients who are predicted to benefit most based on their unique genetic and immunological blueprint.

In addition to addressing genetic knowledge gaps in diverse populations, our fundamental knowledge of T1D progression is limited by the current reliance on animal models, which poses key translational limitations for human pathogenesis. As discussed above, rodent data are not completely applicable to human pathogenesis, and it has been noted that rodent islet histopathology is remarkably different from what has been observed in human samples (244). Therefore, future research must prioritize improvements in longitudinal studies to address differences between rodent and human autoimmunity progression during the asymptomatic first and second phases of T1D pathogenesis. The DiViD study has successfully obtained pancreatic tissue from live T1D patients (48). These efforts must be expanded to include genetically susceptible, AAb+, long-duration, and additional recent-onset T1D patients from more diverse ethnic backgrounds. Follow-up biopsies on these individuals would fill knowledge gaps on the progression of T1D in a human context. Furthermore, adding tissue collection for cell reprogramming into human induced PSCs would allow for robust experiments to answer cell-specific questions that arise in the field, such as better representations of heterogeneity (240–242). Considering this, future studies should focus on improving *ex vivo* and *in vitro* human models, such as SC-islets, to allow for genetic and functional studies that could lead to more effective therapies for T1D.

Beyond methodological advances, a critical area of focus lies in understanding the early stages of T cell development. The existence of monospecific naïve CD8+ T cells in healthy patients suggests a trigger that induces their differentiation into memory and effector T cells in AAb+ and T1D patients (30, 34). Research is necessary to understand the purpose of these default CD8+ T cells. We must determine whether they serve a protective role and the specific events or triggers that cause their shift to a pathogenic, multi-specific state. Deciphering the genetic and environmental triggers that drive their differentiation into stem-like and memory T cells could also offer critical insights for prevention. Human *in vitro* models from AAb+ or T1D patients, such as hPSC-derived cells, will help address these questions. In addition, using organs-on-chips to co-culture pancreatic endothelial cells, immune cells, and islets would highlight the mechanisms of human immune cell infiltration and validate the observations made in animal models. Since loss of CD8+ T cells to cell exhaustion is offset by the presence of stem-like T cells, drug screens targeting these pancreatic stem-like T cells may prove helpful in suppressing disease pathogenesis before clinical diagnosis. These candidates can be used in conjunction with other compounds that further promote CD4+ and CD8+ deactivation via ductal and endothelial cell signaling (20, 35, 245). Overall, furthering our understanding of T cell behavior also requires investigating their response to environmental triggers, particularly viral infections.

The mechanistic effects of CVB infection have been primarily studied in non-pancreatic cell types. Despite reports of β cell Golgi stress and dysregulation of Golgi-associated genes in T1D, as well as the role of the Golgi in class I HLA hypo-expression following CVB infection, the effects of CVB infection on islet Golgi integrity are poorly studied (138, 158, 246). Given the role of cell stress in T1D onset, further studies must also investigate the effects of viral infection in conjunction with other environmental stressors, such as pro-inflammatory cytokines, on β cell health. Moreover, CVB infection may contribute to islet autoimmunity through activated immune responses, but current studies measuring the impact of CVB infection on immune cells have used PBMCs from healthy donors. More recently, Vecchio et al. reported that GAD autoreactive CD8+ T cells derived from T1D PBMCs recognize CVB3 viral peptides (159). However, these experiments were performed on previously frozen PBMCs, which have been shown not to elicit a type I IFN response following CVB3 infection (115). Therefore, further studies with fresh T1D patient-derived PBMCs must be performed to validate these findings and uncover any new insights. Recently, CVB3 was observed to infect all cell types present in isolated primary human islets, including endocrine, exocrine, vascular, and immune cells, and elicit a cell-type-specific transcriptional response using single-cell RNA sequencing (149). However, future studies must functionally investigate cell-to-cell communication and interaction, especially using T1D samples. These efforts are necessary to delineate how CVB infection holistically impacts the pancreatic microenvironment and to find mechanisms linked to T1D pathogenesis.

There is increasing evidence that β cells have an active role in their own destruction during T1D onset, as more than 80% of T1D-associated genes are expressed in β cells (67, 189, 190). Thus far, the molecular effects of *IFIH1* variants associated with T1D have mostly been reported in human PBMCs and mouse models. In these studies, at-risk alleles increased type I IFN response to environmental stress factors, whereas protective alleles reduced this signature (106, 107, 109, 110, 113–115). However, this does not provide any insight into the role of these variants in islet immune response, β cell autoimmunity, β cell health and function, and disease onset. Such studies are difficult to conduct, as β cells from T1D patients have been mostly destroyed, and any isolated cells are of low quality. SC-islets provide an opportunity to generate quality β cells from a T1D background, but such studies remain to be done.

The complexity of T1D, characterized by a multifaceted interplay between genetics and the environment, presents persistent challenges to developing a preventive cure. While significant progress has been made in identifying key genetic loci, the role of the immune system in disease initiation and progression, and understanding basic viral mechanisms, critical knowledge gaps remain. A deeper understanding of the functional role of T1D-associated genes in human-relevant contexts, the intricate dynamics of viral infection within the pancreatic microenvironment, and the specific triggers that shift the immune response from tolerance to autoimmunity is still needed. The future of T1D research hinges on leveraging advanced human models, such as single-cell multiomics, patient-specific data, hPSC-derived cells, and organs-on-chips, to bridge the divide between foundational animal studies and clinical pathology. By focusing on these key areas, the field can move beyond correlational findings to uncover the precise causal mechanisms of T1D, paving the way for targeted interventions that can truly halt disease progression.

## References

[1] Mobasseri M Shirmohammadi M Amiri T Vahed N Fard HH Ghojazadeh M . Prevalence and incidence of type 1 diabetes in the world: A systematic review and meta-analysis. Health Promot Perspect. (2020) 10:98–115. doi: 10.34172/hpp.2020.18, PMID: 32296622 PMC7146037

[2] Shamoon H Duffy H Fleischer N Engel S Saenger P Strelzyn M . The effect of intensive treatment of diabetes on the development and progression of long-term complications in insulin-dependent diabetes mellitus. New Engl J Med. (1993) 329:977–86. doi: 10.1056/NEJM199309303291401, PMID: 8366922

[3] Pickup JC . Insulin-pump therapy for type 1 diabetes mellitus. New Engl J Med. (2012) 366:1616–24. doi: 10.1056/NEJMct1113948, PMID: 22533577

[4] Atkinson MA Eisenbarth GS Michels AW . Type 1 diabetes. Lancet. (2014) 383:69–82. doi: 10.1016/S0140-6736(13)60591-7, PMID: 23890997 PMC4380133

[5] Diagnosis and classification of diabetes mellitus. Diabetes Care. (2014) 37(Supplement 1):S81–90. doi: 10.2337/dc14-S081, PMID: 24357215

[6] Nekoua MP Alidjinou EK Hober D . Persistent coxsackievirus B infection and pathogenesis of type 1 diabetes mellitus. Nat Rev Endocrinol. (2022) 18:503–16. doi: 10.1038/S41574-022-00688-1, PMID: 35650334 PMC9157043

[7] Shapiro MR Thirawatananond P Peters L Sharp RC Ogundare S Posgai AL . De-coding genetic risk variants in type 1 diabetes. Immunol Cell Biol. (2021) 99:496–508. doi: 10.1111/IMCB.12438, PMID: 33483996 PMC8119379

[8] Redondo MJ Jeffrey J Fain PR Eisenbarth GS Orban T . Concordance for islet autoimmunity among monozygotic twins. The New England Journal of Medicine (2008) 359:2849–50. doi: 10.1056/NEJMC0805398, PMID: 19109586

[9] Geravandi S Liu H Maedler K . Enteroviruses and t1d: Is it the virus, the genes or both which cause t1d. Microorganisms. (2020) 8:1–20. doi: 10.3390/microorganisms8071017, PMID: 32650582 PMC7409303

[10] Ilonen J Lempainen J Veijola R . The heterogeneous pathogenesis of type 1 diabetes mellitus. Nat Rev Endocrinol. (2019) 15:635–50. doi: 10.1038/S41574-019-0254-Y, PMID: 31534209

[11] Honkanen H Oikarinen S Nurminen N Laitinen OH Huhtala H Lehtonen J . Detection of enteroviruses in stools precedes islet autoimmunity by several months: possible evidence for slowly operating mechanisms in virus-induced autoimmunity. Diabetologia. (2017) 60:424–31. doi: 10.1007/S00125-016-4177-Z, PMID: 28070615

[12] Stone VM Butrym M Hankaniemi MM Sioofy-Khojine AB Hytönen VP Hyöty H . Coxsackievirus B vaccines prevent infection-accelerated diabetes in NOD mice and have no disease-inducing effect. Diabetes. (2021) 70:2871–8. doi: 10.2337/DB21-0193, PMID: 34497136 PMC8660981

[13] Regnell SE Lernmark Å . Early prediction of autoimmune (type 1) diabetes. Diabetologia. (2017) 60:1370–81. doi: 10.1007/S00125-017-4308-1, PMID: 28550517 PMC5491594

[14] Krischer JP Liu X Hagopian WA Rewers MJ She J-X . Predictors of the initiation of islet autoimmunity and progression to multiple autoantibodies and clinical diabetes: the TEDDY study. Diabetes Care. (2022) 45:2271–81. doi: 10.2337/DC21-2612, PMID: 36150053 PMC9643148

[15] Krischer JP Lynch KF Schatz DA Ilonen J Lernmark Å Hagopian WA . The 6 year incidence of diabetes-associated autoantibodies in genetically at-risk children: the TEDDY study. Diabetologia. (2015) 58:980–7. doi: 10.1007/S00125-015-3514-Y, PMID: 25660258 PMC4393776

[16] Rodriguez-Calvo T Johnson JD Overbergh L Dunne JL . Neoepitopes in type 1 diabetes: etiological insights, biomarkers and therapeutic targets. Front Immunol. (2021) 12:667989. doi: 10.3389/FIMMU.2021.667989, PMID: 33953728 PMC8089389

[17] Roep BO Kracht MJ van Lummel M Zaldumbide A . A roadmap of the generation of neoantigens as targets of the immune system in type 1 diabetes. Curr Opin Immunol. (2016) 43:67–73. doi: 10.1016/J.COI.2016.09.007, PMID: 27723537

[18] Zajec A Trebušak Podkrajšek K Tesovnik T Šket R Čugalj Kern B Jenko Bizjan B . Pathogenesis of type 1 diabetes: established facts and new insights. Genes. (2022) 13:706. doi: 10.3390/GENES13040706, PMID: 35456512 PMC9032728

[19] Bloem SJ Roep BO . The elusive role of B lymphocytes and islet autoantibodies in (human) type 1 diabetes. Diabetologia. (2017) 60:1185–9. doi: 10.1007/S00125-017-4284-5, PMID: 28439640

[20] Fasolino M Schwartz GW Patil AR Mongia A Golson ML Wang YJ . Single-cell multi-omics analysis of human pancreatic islets reveals novel cellular states in type 1 diabetes. Nat Metab. (2022) 4:284–99. doi: 10.1038/s42255-022-00531-x, PMID: 35228745 PMC8938904

[21] Jacobsen LM Diggins K Blanchfield L McNichols J Perry DJ Brant J . Responders to low-dose ATG induce CD4+ T cell exhaustion in type 1 diabetes. JCI Insight. (2023) 8:e161812. doi: 10.1172/JCI.INSIGHT.161812, PMID: 37432736 PMC10543726

[22] Long SA Thorpe J DeBerg HA Gersuk V Eddy JA Harris KM . Linsley PS. Partial exhaustion of CD8 T cells and clinical response to teplizumab in new-onset type 1 diabetes. Sci Immunol. (2016) 1. doi: 10.1126/SCIIMMUNOL.AAI7793, PMID: 28664195 PMC5486405

[23] Tooley JE Vudattu N Choi J Cotsapas C Devine L Raddassi K . Changes in T-cell subsets identify responders to FcR-nonbinding anti-CD3 mAb (teplizumab) in patients with type 1 diabetes. Eur J Immunol. (2016) 46:230–41. doi: 10.1002/EJI.201545708, PMID: 26518356 PMC4882099

[24] Sakaguchi S Sakaguchi N Asano M Itoh M Toda M . Immunologic self-tolerance maintained by activated T cells expressing IL-2 receptor α-chains (CD25). Breakdown of a single mechanism of self-tolerance causes various autoimmune diseases. J Immunol. (1995) 155:1151–64. doi: 10.4049/JIMMUNOL.155.3.1151 7636184

[25] Alhazmi A Nekoua MP Michaux H Sane F Halouani A Engelmann I . Effect of coxsackievirus b4 infection on the thymus: Elucidating its role in the pathogenesis of type 1 diabetes. Microorganisms. (2021) 9. doi: 10.3390/microorganisms9061177, PMID: 34072590 PMC8229779

[26] Geenen V . The thymus and the science of self. Semin Immunopathol. (2021) 43:5–14. doi: 10.1007/S00281-020-00831-Y, PMID: 33415360 PMC7925502

[27] Anindya R Rutter GA Meur G . New-onset type 1 diabetes and severe acute respiratory syndrome coronavirus 2 infection. Immunol Cell Biol. (2023) 101:191–203. doi: 10.1111/IMCB.12615, PMID: 36529987 PMC9877852

[28] Wculek SK Cueto FJ Mujal AM Melero I Krummel MF Sancho D . Dendritic cells in cancer immunology and immunotherapy. Nat Rev Immunol. (2019) 20:7–24. doi: 10.1038/s41577-019-0210-z, PMID: 31467405

[29] Rodriguez-Calvo T Ekwall O Amirian N Zapardiel-Gonzalo J Von Herrath MG . Increased immune cell infiltration of the exocrine pancreas: A possible contribution to the pathogenesis of type 1 diabetes. Diabetes. (2014) 63:3880–90. doi: 10.2337/DB14-0549, PMID: 24947367 PMC4207385

[30] Landry LG Anderson AM Russ HA Yu L Kent SC Atkinson MA . Proinsulin-reactive CD4 T cells in the islets of type 1 diabetes organ donors. Front Endocrinol (Lausanne). (2021) 12:622647. doi: 10.3389/FENDO.2021.622647, PMID: 33841327 PMC8027116

[31] Schnell A . Stem-like T cells in cancer and autoimmunity. Immunol Rev. (2024) 325:9–22. doi: 10.1111/IMR.13356, PMID: 38804499

[32] Ahrends T Spanjaard A Pilzecker B Bąbała N Bovens A Xiao Y . CD4+ T cell help confers a cytotoxic T cell effector program including coinhibitory receptor downregulation and increased tissue invasiveness. Immunity. (2017) 47:848–861.e5. doi: 10.1016/j.immuni.2017.10.009, PMID: 29126798

[33] Lu YJ Barreira-Silva P Boyce S Powers J Cavallo K Behar SM . CD4 T cell help prevents CD8 T cell exhaustion and promotes control of Mycobacterium tuberculosis infection. Cell Rep. (2021) 36:109696. doi: 10.1016/J.CELREP.2021.109696, PMID: 34525366 PMC8466141

[34] Bender C Rodriguez-Calvo T Amirian N Coppieters KT von Herrath MG . The healthy exocrine pancreas contains preproinsulin-specific CD8 T cells that attack islets in type 1 diabetes. Sci Adv. (2020) 6:5586–602. doi: 10.1126/SCIADV.ABC5586, PMID: 33067232 PMC7567597

[35] Barlow GL Schürch CM Bhate SS Phillips DJ Young A Dong S . The extra-islet pancreas supports autoimmunity in human type 1 diabetes. Elife. (2025) 13. doi: 10.7554/ELIFE.100535, PMID: 40232951 PMC11999700

[36] Kuric E Seiron P Krogvold L Edwin B Buanes T Hanssen KF . Demonstration of tissue resident memory CD8 T cells in insulitic lesions in adult patients with recent-onset type 1 diabetes. Am J Pathol. (2017) 187:581–8. doi: 10.1016/J.AJPATH.2016.11.002, PMID: 28212742

[37] Vignali D Cantarelli E Bordignon C Canu A Citro A Annoni A . Detection and characterization of CD8+ Autoreactive memory stem T cells in patients with type 1 diabetes. Diabetes. (2018) 67:936–45. doi: 10.2337/DB17-1390, PMID: 29506985

[38] Gearty SV Dündar F Zumbo P Espinosa-Carrasco G Shakiba M Sanchez-Rivera FJ . An autoimmune stem-like CD8 T cell population drives type 1 diabetes. Nature. (2021) 602:156. doi: 10.1038/S41586-021-04248-X, PMID: 34847567 PMC9315050

[39] Anderson AM Landry LG Alkanani AA Pyle L Powers AC Atkinson MA . Human islet T cells are highly reactive to preproinsulin in type 1 diabetes. Proc Natl Acad Sci U.S.A. (2021) 118:e2107208118. doi: 10.1073/PNAS.2107208118, PMID: 34611019 PMC8521679

[40] Savinov AY Wong FS Stonebraker AC Chervonsky AV . Presentation of antigen by endothelial cells and chemoattraction are required for homing of insulin-specific CD8+ T cells. J Exp Med. (2003) 197:643–56. doi: 10.1084/JEM.20021378, PMID: 12615905 PMC2193823

[41] Coppieters KT Dotta F Amirian N Campbell PD Kay TWH Atkinson MA . Demonstration of islet-autoreactive CD8 T cells in insulitic lesions from recent onset and long-term type 1 diabetes patients. J Exp Med. (2012) 209:51–60. doi: 10.1084/JEM.20111187, PMID: 22213807 PMC3260877

[42] Richardson SJ Rodriguez-Calvo T Gerling IC Mathews CE Kaddis JS Russell MA . Islet cell hyperexpression of HLA class I antigens: a defining feature in type 1 diabetes. Diabetologia. (2016) 59:2448–58. doi: 10.1007/S00125-016-4067-4, PMID: 27506584 PMC5042874

[43] Bogdani M Korpos E Simeonovic CJ Parish CR Sorokin L Wight TN . Extracellular matrix components in the pathogenesis of type 1 diabetes. Curr Diabetes Rep. (2014) 14:1–11. doi: 10.1007/s11892-014-0552-7, PMID: 25344787 PMC4238291

[44] Korpos É Kadri N Kappelhoff R Wegner J Overall CM Weber E . The peri-islet basement membrane, a barrier to infiltrating leukocytes in type 1 diabetes in mouse and human. Diabetes. (2013) 62:531–42. doi: 10.2337/DB12-0432/-/DC1 PMC3554379 23139348

[45] Martins CP New LA O’Connor EC Previte DM Cargill KR Tse IL . Glycolysis inhibition induces functional and metabolic exhaustion of CD4+ T cells in type 1 diabetes. Front Immunol. (2021) 12:669456. doi: 10.3389/FIMMU.2021.669456, PMID: 34163475 PMC8216385

[46] Demine S Schiavo AA Marín-Cañas S Marchetti P Cnop M Eizirik DL . Pro-inflammatory cytokines induce cell death, inflammatory responses, and endoplasmic reticulum stress in human iPSC-derived beta cells. Stem Cell Res Ther. (2020) 11. doi: 10.1186/s13287-019-1523-3, PMID: 31900242 PMC6942385

[47] Campbell-Thompson ML Atkinson MA Butler AE Chapman NM Frisk G Gianani R . The diagnosis of insulitis in human type 1 diabetes. Diabetologia. (2013) 56:2541–3. doi: 10.1007/S00125-013-3043-5, PMID: 24006089

[48] Krogvold L Edwin B Buanes T Frisk G Skog O Anagandula M . Detection of a low-grade enteroviral infection in the islets of langerhans of living patients newly diagnosed with type 1 diabetes. Diabetes. (2015) 64:1682–7. doi: 10.2337/db14-1370, PMID: 25422108

[49] In’t Veld P . Insulitis in human type 1 diabetes: The quest for an elusive lesion. Islets. (2011) 3:131–8. doi: 10.4161/ISL.3.4.15728, PMID: 21606672 PMC3154446

[50] Gootjes C Zwaginga JJ Roep BO Nikolic T . Functional impact of risk gene variants on the autoimmune responses in type 1 diabetes. Front Immunol. (2022) 13:886736. doi: 10.3389/FIMMU.2022.886736, PMID: 35603161 PMC9114814

[51] Harrison JW Tallapragada DSP Baptist A Sharp SA Bhaskar S Jog KS . Type 1 diabetes genetic risk score is discriminative of diabetes in non-Europeans: evidence from a study in India. Sci Rep. (2020) 10:1–7. doi: 10.1038/s41598-020-65317-1, PMID: 32528078 PMC7289794

[52] Sharp SA Rich SS Wood AR Jones SE Beaumont RN Harrison JW . Development and standardization of an improved type 1 diabetes genetic risk score for use in newborn screening and incident diagnosis. Diabetes Care. (2019) 42:200–7. doi: 10.2337/DC18-1785, PMID: 30655379 PMC6341291

[53] Patel KA Oram RA Flanagan SE De Franco E Colclough K Shepherd M . Type 1 diabetes genetic risk score: A novel tool to discriminate monogenic and type 1 diabetes. Diabetes. (2016) 65:2094–9. doi: 10.2337/DB15-1690, PMID: 27207547 PMC4920219

[54] Chiou J Geusz RJ Okino ML Han JY Miller M Melton R . Interpreting type 1 diabetes risk with genetics and single cell epigenomics. Nature. (2021) 594:398. doi: 10.1038/S41586-021-03552-W, PMID: 34012112 PMC10560508

[55] Vahedi G Kanno Y Furumoto Y Jiang K Parker SCJ Erdos MR . Super-enhancers delineate disease-associated regulatory nodes in T cells. Nature. (2015) 520:558–62. doi: 10.1038/nature14154, PMID: 25686607 PMC4409450

[56] Onengut-Gumuscu S Chen WM Burren O Cooper NJ Quinlan AR Mychaleckyj JC . Fine mapping of type 1 diabetes susceptibility loci and evidence for colocalization of causal variants with lymphoid gene enhancers. Nat Genet. (2015) 47:381–6. doi: 10.1038/ng.3245, PMID: 25751624 PMC4380767

[57] Mittal R Camick N Lemos JRN Hirani K . Gene-environment interaction in the pathophysiology of type 1 diabetes. Front Endocrinol (Lausanne). (2024) 15:1335435. doi: 10.3389/FENDO.2024.1335435, PMID: 38344660 PMC10858453

[58] Koeleman BPC Lie BA Undlien DE Dudbridge F Thorsby E de Vries RRP . Genotype effects and epistasis in type 1 diabetes and HLA-DQ trans dimer associations with disease. Genes Immun. (2004) 5:381–8. doi: 10.1038/SJ.GENE.6364106, PMID: 15164102

[59] Luckett AM Weedon MN Hawkes G Leslie RD Oram RA Grant SFA . Utility of genetic risk scores in type 1 diabetes. Diabetologia. (2023) 66:1589–600. doi: 10.1007/S00125-023-05955-Y, PMID: 37439792 PMC10390619

[60] Walldén J Ilonen J Roivainen M Ludvigsson J Vaarala O . Effect of HLA genotype or CTLA-4 polymorphism on cytokine response in healthy children. Scand J Immunol. (2008) 68:345–50. doi: 10.1111/J.1365-3083.2008.02144.X, PMID: 18782261

[61] Noble JA Valdes AM Thomson G Erlich HA . The HLA class II locus DPB1 can influence susceptibility to type 1 diabetes. Diabetes. (2000) 49. Available online at: http://diabetesjournals.org/diabetes/article-pdf/49/1/121/364678/10615959.pdf (Accessed July 5, 2025)., PMID: 10615959 10.2337/diabetes.49.1.121

[62] Törn C Hadley D Lee HS Hagopian W Lernmark Å Simell O . Role of type 1 diabetes–associated SNPs on risk of autoantibody positivity in the TEDDY study. Diabetes. (2015) 64:1818–29. doi: 10.2337/DB14-1497, PMID: 25422107 PMC4407865

[63] Pugliese A Zeller M Fernandez A Zalcberg LJ Bartlett RJ Ricordi C . The insulin gene is transcribed in the human thymus and transcription levels correlate with allelic variation at the INS VNTR-IDDM2 susceptibility locus for type 1 diabetes. Nat Genet. (1997) 15:293–7. doi: 10.1038/NG0397-293, PMID: 9054945

[64] Vafiadis P Bennett ST Todd JA Nadeau J Grabs R Goodyer CG . Insulin expression in human thymus is modulated by INS VNTR alleles at the IDDM2 locus. Nat Genet. (1997) 15:289–92. doi: 10.1038/NG0397-289, PMID: 9054944

[65] Nielsen LB Mortensen HB Chiarelli F Holl R Swift P De Beaufort C . Impact of IDDM2 on disease pathogenesis and progression in children with newly diagnosed type 1 diabetes: Reduced insulin antibody titres and preserved beta cell function. Diabetologia. (2006) 49:71–4. doi: 10.1007/S00125-005-0042-1, PMID: 16307231

[66] van Tienhoven R Vu AN Kaddis JS Roep BO . Low risk for diabetic complications in type 1 diabetes patients carrying a protective insulin gene variant. PloS One. (2023) 18:e0280872. doi: 10.1371/JOURNAL.PONE.0280872, PMID: 36701305 PMC9879388

[67] Marroqui L Dos Santos RS Fløyel T Grieco FA Santin I Op De Beeck A . TYK2, a candidate gene for type 1 diabetes, modulates apoptosis and the innate immune response in human pancreatic β-cells. Diabetes. (2015) 64:3808–17. doi: 10.2337/DB15-0362, PMID: 26239055

[68] Robertson CC Inshaw JRJ Onengut-Gumuscu S Chen WM Santa Cruz DF Yang H . Fine-mapping, trans-ancestral and genomic analyses identify causal variants, cells, genes and drug targets for type 1 diabetes. Nat Genet. (2021) 53:962–71. doi: 10.1038/s41588-021-00880-5, PMID: 34127860 PMC8273124

[69] Bottini N Musumeci L Alonso A Rahmouni S Nika K Rostamkhani M . A functional variant of lymphoid tyrosine phosphatase is associated with type I diabetes. Nat Genet. (2004) 36:337–8. doi: 10.1038/NG1323, PMID: 15004560

[70] Fiorillo E Orrú V Stanford SM Liu Y Salek M Rapini N . Autoimmune-associated PTPN22 R620W variation reduces phosphorylation of lymphoid phosphatase on an inhibitory tyrosine residue. J Biol Chem. (2010) 285:26506–18. doi: 10.1074/JBC.M110.111104, PMID: 20538612 PMC2924087

[71] Sharp RC Beg SA Naser SA . Polymorphisms in protein Tyrosine Phosphatase Non-receptor type 2 and 22 (PTPN2/22) are linked to hyper-proliferative T-cells and susceptibility to Mycobacteria in Rheumatoid arthritis. Front Cell Infect Microbiol. (2018) 8:11. doi: 10.3389/FCIMB.2018.00011, PMID: 29423382 PMC5788942

[72] Vang T Congia M Macis MD Musumeci L Orrú V Zavattari P . Autoimmune-associated lymphoid tyrosine phosphatase is a gain-of-function variant. Nat Genet. (2005) 37:1317–9. doi: 10.1038/NG1673, PMID: 16273109

[73] Perry DJ Peters LD Lakshmi PS Zhang L Han Z Wasserfall CH . Overexpression of the PTPN22 autoimmune risk variant LYP-620W fails to restrain human CD4+ T cell activation. J Immunol. (2021) 207:849–59. doi: 10.4049/JIMMUNOL.2000708, PMID: 34301848 PMC8323970

[74] Anderson W Barahmand-Pour-Whitman F Linsley PS Cerosaletti K Buckner JH Rawlings DJ . PTPN22 R620W gene editing in T cells enhances low-avidity TCR responses. Elife. (2023) 12. doi: 10.7554/ELIFE.81577, PMID: 36961507 PMC10065793

[75] Valta M Gazali AM Viisanen T Ihantola EL Ekman I Toppari J . Type 1 diabetes linked PTPN22 gene polymorphism is associated with the frequency of circulating regulatory T cells. Eur J Immunol. (2020) 50:581–8. doi: 10.1002/EJI.201948378, PMID: 31808541

[76] Aarnisalo J Treszl A Svec P Marttila J Öling V Simell O . Reduced CD4+T cell activation in children with type 1 diabetes carrying the PTPN22/Lyp 620Trp variant. J Autoimmun. (2008) 31:13–21. doi: 10.1016/J.JAUT.2008.01.001, PMID: 18299186

[77] Ge Y Paisie TK Newman JRB McIntyre LM Concannon P . UBASH3A mediates risk for type 1 diabetes through inhibition of T-cell receptor–induced NF-κB signaling. Diabetes. (2017) 66:2033–43. doi: 10.2337/DB16-1023, PMID: 28607106 PMC5482087

[78] Newman JRB Concannon P Ge Y . UBASH3A interacts with PTPN22 to regulate IL2 expression and risk for type 1 diabetes. Int J Mol Sci. (2023) 24:8671. doi: 10.3390/IJMS24108671, PMID: 37240014 PMC10218450

[79] Ge Y Concannon P . Molecular-genetic characterization of common, noncoding UBASH3A variants associated with type 1 diabetes. Eur J Hum Genet. (2018) 26:1060–4. doi: 10.1038/S41431-018-0123-5, PMID: 29491471 PMC6018660

[80] Chen YG Ciecko AE Khaja S Grzybowski M Geurts AM Lieberman SM . UBASH3A deficiency accelerates type 1 diabetes development and enhances salivary gland inflammation in NOD mice. Sci Rep. (2020) 10:1–12. doi: 10.1038/S41598-020-68956-6, PMID: 32694640 PMC7374577

[81] Mordes JP Cort L Pierce BG Liu Z Eberwine R . Blankenhorn EP. T cell receptor genotype and ubash3a determine susceptibility to rat autoimmune diabetes. Genes. (2021) 12:852. doi: 10.3390/GENES12060852, PMID: 34205929 PMC8227067

[82] Sharp RC Brown ME Shapiro MR Posgai AL Brusko TM . The immunoregulatory role of the signal regulatory protein family and CD47 signaling pathway in type 1 diabetes. Front Immunol. (2021) 12:739048/REFERENCE. doi: 10.3389/FIMMU.2021.739048/REFERENCE, PMID: 34603322 PMC8481641

[83] Smith MJ Pastor L Newman JRB Concannon P . Genetic control of splicing at SIRPG modulates risk of type 1 diabetes. Diabetes. (2022) 71:350–8. doi: 10.2337/DB21-0194, PMID: 34799406 PMC8914281

[84] Sinha S Borcherding N Renavikar PS Crawford MP Tsalikian E Tansey M . An autoimmune disease risk SNP, rs2281808, in SIRPG is associated with reduced expression of SIRPγ and heightened effector state in human CD8 T-cells. Sci Rep. (2018) 8:1–9. doi: 10.1038/s41598-018-33901-1, PMID: 30337675 PMC6194019

[85] Hossen MM Ma Y Yin Z Xia Y Du J Huang JY . Current understanding of CTLA-4: from mechanism to autoimmune diseases. Front Immunol. (2023) 14:1198365/XML. doi: 10.3389/FIMMU.2023.1198365/XML, PMID: 37497212 PMC10367421

[86] Wing K Onishi Y Prieto-Martin P Yamaguchi T Miyara M Fehervari Z . CTLA-4 control over Foxp3+ regulatory T cell function. Sci (1979). (2008) 322:271–5. doi: 10.1126/SCIENCE.1160062, PMID: 18845758

[87] You S Alyanakian MA Segovia B Damotte D Bluestone J Bach JF . Immunoregulatory pathways controlling progression of autoimmunity in NOD mice: The role of CTLA-4 and TGF-β. Ann N Y Acad Sci. (2008) 1150:300–10. doi: 10.1196/ANNALS.1447.046, PMID: 19120317 PMC3087168

[88] Fujii M Inoguchi T Batchuluun B Sugiyama N Kobayashi K Sonoda N . CTLA-4Ig immunotherapy of obesity-induced insulin resistance by manipulation of macrophage polarization in adipose tissues. Biochem Biophys Res Commun. (2013) 438:103–9. doi: 10.1016/J.BBRC.2013.07.034, PMID: 23872146

[89] Balic I Angel B Codner E Carrasco E Pérez-Bravo F . Association of CTLA-4 polymorphisms and clinical-immunologic characteristics at onset of type 1 diabetes mellitus in children. Hum Immunol. (2009) 70:116–20. doi: 10.1016/J.HUMIMM.2008.12.007, PMID: 19136037

[90] Anjos S Nguyen A Ounissi-Benkalha H Tessier MC Polychronakos C . A common autoimmunity predisposing signal peptide variant of the cytotoxic T-lymphocyte antigen 4 results in inefficient glycosylation of the susceptibility allele. J Biol Chem. (2002) 277:46478–86. doi: 10.1074/JBC.M206894200, PMID: 12244107

[91] Chen Y Chen S Gu Y Feng Y Shi Y Fu Q . CTLA-4 + 49 G/A, a functional T1D risk SNP, affects CTLA-4 level in Treg subsets and IA-2A positivity, but not beta-cell function. Sci Rep. (2018) 8:1–8. doi: 10.1038/s41598-018-28423-9, PMID: 29973665 PMC6031668

[92] Liu K Liu Y Xiong H Ning Z . The immune regulatory functions of CD226 and its implications in immune-mediated diseases. Biomolecules. (2025) 15:1007. doi: 10.3390/BIOM15071007, PMID: 40723878 PMC12292313

[93] Gaud G Roncagalli R Chaoui K Bernard I Familiades J Colacios C . The costimulatory molecule CD226 signals through VAV1 to amplify TCR signals and promote IL-17 production by CD4+ T cells. Sci Signal. (2018) 11:3083. doi: 10.1126/SCISIGNAL.AAR3083, PMID: 29991650

[94] Shapiro MR Yeh WI Longfield JR Gallagher J Infante CM Wellford S . CD226 deletion reduces type 1 diabetes in the NOD mouse by impairing thymocyte development and peripheral T cell activation. Front Immunol. (2020) 11:2180. doi: 10.3389/FIMMU.2020.02180, PMID: 33013915 PMC7500101

[95] Thirawatananond P Brown ME Sachs LK Arnoletti JM Yeh WI Posgai AL . Treg-specific CD226 deletion reduces diabetes incidence in NOD mice by improving regulatory T-cell stability. Diabetes. (2023) 72:1629–40. doi: 10.2337/DB23-0307, PMID: 37625150 PMC10588280

[96] Brown ME Thirawatananond P Peters LD Kern EJ Vijay S Sachs LK . Inhibition of CD226 co-stimulation suppresses diabetes development in the NOD mouse by augmenting regulatory T cells and diminishing effector T cell function. Diabetologia. (2025) 68:397–418. doi: 10.1007/s00125-024-06329-8, PMID: 39636437 PMC11732877

[97] Zhong T Li X Lei K Tang R Deng Q Love PE . TGF-β-mediated crosstalk between TIGIT+ Tregs and CD226+CD8+ T cells in the progression and remission of type 1 diabetes. Nat Commun. (2024) 15:1–18. doi: 10.1038/s41467-024-53264-8, PMID: 39406740 PMC11480485

[98] Wang JP Cerny A Asher DR Kurt-Jones EA Bronson RT Finberg RW . MDA5 and MAVS mediate type I interferon responses to coxsackie B virus. J Virol. (2010) 84:254–60. doi: 10.1128/JVI.00631-09, PMID: 19846534 PMC2798442

[99] Dias Junior AG Sampaio NG Rehwinkel J . A balancing act: MDA5 in antiviral immunity and autoinflammation. Trends Microbiol. (2019) 27:75–85. doi: 10.1016/j.tim.2018.08.007, PMID: 30201512 PMC6319154

[100] Kato H Takeuchi O Mikamo-Satoh E Hirai R Kawai T Matsushita K . Length-dependent recognition of double-stranded ribonucleic acids by retinoic acid–inducible gene-I and melanoma differentiation–associated gene 5. J Exp Med. (2008) 205:1601–10. doi: 10.1084/JEM.20080091, PMID: 18591409 PMC2442638

[101] Peisley A Lin C Wu B Orme-Johnson M Liu M Walz T . Cooperative assembly and dynamic disassembly of MDA5 filaments for viral dsRNA recognition. Proc Natl Acad Sci U.S.A. (2011) 108:21010–5. doi: 10.1073/PNAS.1113651108, PMID: 22160685 PMC3248507

[102] Peisley A Jo MH Lin C Wu B Orme-Johnson M Walz T . Kinetic mechanism for viral dsRNA length discrimination by MDA5 filaments. Proc Natl Acad Sci U.S.A. (2012) 109:E3340–9. doi: 10.1073/PNAS.1208618109, PMID: 23129641 PMC3523859

[103] Wu B Peisley A Richards C Yao H Zeng X Lin C . Structural basis for dsRNA recognition, filament formation, and antiviral signal activation by MDA5. Cell. (2013) 152:276–89. doi: 10.1016/j.cell.2012.11.048, PMID: 23273991

[104] Liu G Lee J-H✉ Lee J-H Parker ZM Acharya D Chiang JJ . ISG15-dependent activation of the sensor MDA5 is antagonized by the SARS-CoV-2 papain-like protease to evade host innate immunity. Nat Microbiol. (2021) 6:467–78. doi: 10.1038/s41564-021-00884-1, PMID: 33727702 PMC8103894

[105] Pociot F . Type 1 diabetes genome-wide association studies: not to be lost in translation. Clin Transl Immunol. (2017) 6:e162. doi: 10.1038/CTI.2017.51, PMID: 29333267 PMC5750451

[106] Downes K Pekalski M Angus KL Hardy M Nutland S Smyth DJ . Reduced expression of IFIH1 is protective for type 1 diabetes. PloS One. (2010) 5:1–6. doi: 10.1371/journal.pone.0012646, PMID: 20844740 PMC2936573

[107] Chistiakov DA Voronova NV Savost’Anov KV Turakulov RI . Loss-of-function mutations E6 27X and I923V of IFIH1 are associated with lower poly(I:C)-induced interferon-β production in peripheral blood mononuclear cells of type 1 diabetes patients. Hum Immunol. (2010) 71:1128–34. doi: 10.1016/J.HUMIMM.2010.08.005, PMID: 20736039

[108] Shigemoto T Kageyama M Hirai R Zheng JP Yoneyama M Fujita T . Identification of loss of function mutations in human genes encoding RIG-I and MDA5: IMPLICATIONS FOR RESISTANCE TO TYPE I DIABETES. J Biol Chem. (2009) 284:13348–54. doi: 10.1074/JBC.M809449200, PMID: 19324880 PMC2679434

[109] Blum SI Taylor JP Barra JM Burg AR Shang Q Qiu S . MDA5-dependent responses contribute to autoimmune diabetes progression and hindrance. JCI Insight. (2023) 8:e157929. doi: 10.1172/JCI.INSIGHT.157929, PMID: 36512407 PMC9977297

[110] Lincez PJ Shanina I Horwitz MS . Reduced expression of the MDA5 gene IFIH1 prevents autoimmune diabetes. Diabetes. (2015) 64:2184–93. doi: 10.2337/DB14-1223, PMID: 25591872

[111] Zervou MI Andreou AC Eliopoulos EE Goulielmos GN . Functional significance of the rare rs35667974 IFIH1 gene polymorphism, associated with multiple autoimmune diseases, using a structural biological approach. Autoimmunity. (2022) 55:455–61. doi: 10.1080/08916934.2022.2103799, PMID: 35918839

[112] Smyth DJ Cooper JD Bailey R Field S Burren O Smink LJ . A genome-wide association study of nonsynonymous SNPs identifies a type 1 diabetes locus in the interferon-induced helicase (IFIH1) region. Nat Genet. (2006) 38:617–9. doi: 10.1038/NG1800, PMID: 16699517

[113] Gorman JA Hundhausen C Errett JS Stone AE Allenspach EJ Ge Y . The A946T variant IFIH1 RNA sensor mediates an interferon program that limits viral infection but increases the risk for autoimmunity. Nat Immunol. (2017) 18:744. doi: 10.1038/NI.3766, PMID: 28553952 PMC5697900

[114] Stock AJ Gonzalez Paredes P de Almeida LP Kosanke SD Chetlur S Budde H . The IFIH1-A946T risk variant promotes diabetes in a sex-dependent manner. Front Immunol. (2024) 15:1349601. doi: 10.3389/FIMMU.2024.1349601, PMID: 38487540 PMC10937421

[115] Taylor JP Blum SI Graffeo HC Shang Q Qiu S Green TJ . The type 1 diabetes–associated single nucleotide polymorphism rs1990760 in IFIH1 is associated with increased basal type I IFNs and IFN-stimulated gene expression. J Immunol. (2024) 213:1415–28. doi: 10.4049/JIMMUNOL.2400344, PMID: 39373578 PMC12803751

[116] Ferreira RC Guo H Coulson RMR Smyth DJ Pekalski ML Burren OS . A type I Interferon transcriptional signature precedes autoimmunity in children genetically at risk for type 1 diabetes. Diabetes. (2014) 63:2538–50. doi: 10.2337/db13-1777, PMID: 24561305 PMC4066333

[117] Kallionpää H Elo LL Laajala E Mykkänen J Ricaño-Ponce I Vaarma M . Innate immune activity is detected prior to seroconversion in children with HLA-conferred type 1 diabetes susceptibility. Diabetes. (2014) 63:2402–14. doi: 10.2337/DB13-1775, PMID: 24550192

[118] Russell MA Richardson SJ Morgan NG . The role of the interferon/JAK-STAT axis in driving islet HLA-I hyperexpression in type 1 diabetes. Front Endocrinol (Lausanne). (2023) 14:1270325. doi: 10.3389/FENDO.2023.1270325, PMID: 37867531 PMC10588626

[119] Marroqui L Dos Santos RS Op de beeck A Coomans de Brachène A Marselli L Marchetti P . Interferon-α mediates human beta cell HLA class I overexpression, endoplasmic reticulum stress and apoptosis, three hallmarks of early human type 1 diabetes. Diabetologia. (2017) 60:656–67. doi: 10.1007/S00125-016-4201-3, PMID: 28062922

[120] Li Q Xu B Michie SA Rubins KH Schreriber RD McDevitt HO . Interferon-α initiates type 1 diabetes in nonobese diabetic mice. Proc Natl Acad Sci U.S.A. (2008) 105:12439–44. doi: 10.1073/pnas.0806439105, PMID: 18716002 PMC2527930

[121] Dendrou CA Cortes A Shipman L Evans HG Attfield KE Jostins L . Resolving TYK2 locus genotype-To-phenotype differences in autoimmunity. Sci Transl Med. (2016) 8. doi: 10.1126/SCITRANSLMED.AAG1974, PMID: 27807284 PMC5737835

[122] Mine K Nagafuchi S Akazawa S Abiru N Mori H Kurisaki H . TYK2 signaling promotes the development of autoreactive CD8+ cytotoxic T lymphocytes and type 1 diabetes. Nat Commun. (2024) 15:1–14. doi: 10.1038/s41467-024-45573-9, PMID: 38351043 PMC10864272

[123] Syed F Ballew O Lee CC Rana J Krishnan P Castela A . Pharmacological inhibition of tyrosine protein-kinase 2 reduces islet inflammation and delays type 1 diabetes onset in mice. EBioMedicine. (2025) 117:105734. doi: 10.1016/J.EBIOM.2025.105734, PMID: 40335415 PMC12173048

[124] Izumi K Mine K Inoue Y Teshima M Ogawa S Kai Y . Reduced Tyk2 gene expression in β-cells due to natural mutation determines susceptibility to virus-induced diabetes. Nat Commun. (2015) 6:1–10. doi: 10.1038/ncomms7748, PMID: 25849081 PMC4396380

[125] Hao H Eberand BM Larance M Haltiwanger RS . Protein O-fucosyltransferases: biological functions and molecular mechanisms in mammals. Molecules. (2025) 30:1470. doi: 10.3390/MOLECULES30071470, PMID: 40286076 PMC11990869

[126] Giampaoli O Conta G Calvani R Miccheli A . Can the FUT2 non-secretor phenotype associated with gut microbiota increase the children susceptibility for type 1 diabetes? A mini review. Front Nutr. (2020) 7:606171. doi: 10.3389/FNUT.2020.606171, PMID: 33425974 PMC7785815

[127] Smyth DJ Cooper JD Howson JMM Clarke P Downes K Mistry T . FUT2 nonsecretor status links type 1 diabetes susceptibility and resistance to infection. Diabetes. (2011) 60:3081–4. doi: 10.2337/DB11-0638, PMID: 22025780 PMC3198057

[128] Wacklin P Tuimala J Nikkilä J Tims S Mäkivuokko H Alakulppi N . Faecal microbiota composition in adults is associated with the FUT2 gene determining the secretor status. PloS One. (2014) 9:e94863. doi: 10.1371/JOURNAL.PONE.0094863, PMID: 24733310 PMC3986271

[129] Azad MB Wade KH Timpson NJ . FUT2 secretor genotype and susceptibility to infections and chronic conditions in the ALSPAC cohort. Wellcome Open Res. (2018) 3:65. doi: 10.12688/WELLCOMEOPENRES.14636.2, PMID: 30345375 PMC6171556

[130] Rewers M Ludvigsson J . Environmental risk factors for type 1 diabetes. Lancet. (2016) 387:2340. doi: 10.1016/S0140-6736(16)30507-4, PMID: 27302273 PMC5571740

[131] Bosi E Molteni L Radaelli MG Folini L Fermo I Bazzigaluppi E . Increased intestinal permeability precedes clinical onset of type 1 diabetes. Diabetologia. (2006) 49:2824–7. doi: 10.1007/S00125-006-0465-3, PMID: 17028899

[132] Watad A Azrielant S Bragazzi NL Sharif K David P Katz I . Seasonality and autoimmune diseases: The contribution of the four seasons to the mosaic of autoimmunity. J Autoimmun. (2017) 82:13–30. doi: 10.1016/j.jaut.2017.06.001, PMID: 28624334

[133] Moltchanova EV Schreier N Lammi N Karvonen M . Seasonal variation of diagnosis of Type 1 diabetes mellitus in children worldwide. Diabetic Med. (2009) 26:673–8. doi: 10.1111/j.1464-5491.2009.02743.x, PMID: 19573115

[134] Barrett-Connor E . Is insulin-dependent diabetes mellitus caused by coxsackievirus B infection? A review of the epidemiologic evidence. Rev Infect Dis. (1985) 7:207–15. doi: 10.1093/CLINIDS/7.2.207, PMID: 2988099

[135] Garmaroudi FS Marchant D Hendry R Luo H Yang D Ye X . Coxsackievirus B3 replication and pathogenesis. Future Microbiol. (2015) 10:629–52. doi: 10.2217/fmb.15.5, PMID: 25865198

[136] Hyypiä T Kallajoki M Maaronen M Stanway G Kandolf R Auvinen P . Pathogenetic differences between coxsackie A and B virus infections in newborn mice. Virus Res. (1993) 27:71–8. doi: 10.1016/0168-1702(93)90113-2, PMID: 8383395

[137] Huber S Ramsingh AI . Review coxsackievirus-induced pancreatitis. Viral Immunology (2004) 17:358–69. doi: 10.1089/vim.2004.17.358, PMID: 15357902

[138] Cornell CT Kiosses WB Harkins S Whitton JL . Inhibition of protein trafficking by coxsackievirus B3: multiple viral proteins target a single organelle. J Virol. (2006) 80:6637–47. doi: 10.1128/jvi.02572-05, PMID: 16775351 PMC1488957

[139] Françozo MCS Costa FRC Guerra-Gomes IC Silva JS Sesti-Costa R . Dendritic cells and regulatory T cells expressing CCR4 provide resistance to coxsackievirus B5-induced pancreatitis. Sci Rep. (2019) 9:1–11. doi: 10.1038/s41598-019-51311-9, PMID: 31611578 PMC6791842

[140] Chehadeh W Kerr-Conte J Pattou F Alm G Lefebvre J Wattré P . Persistent infection of human pancreatic islets by coxsackievirus B is associated with alpha interferon synthesis in β Cells. J Virol. (2000) 74:10153–64. doi: 10.1128/JVI.74.21.10153-10164.2000, PMID: 11024144 PMC102054

[141] Roivainen M Rasilainen S Ylipaasto P Nissinen R Ustinov J Bouwens L . Mechanisms of coxsackievirus-induced damage to human pancreaticβ -cells. J Clin Endocrinol Metab. (2000) 85:432–40. doi: 10.1210/JCEM.85.1.6306, PMID: 10634421

[142] Brilot F Chehadeh W Charlet-Renard C Martens H Geenen V Hober D . Persistent infection of human thymic epithelial cells by coxsackievirus B4. J Virol. (2002) 76:5260–5. doi: 10.1128/JVI.76.10.5260-5265.2002, PMID: 11967339 PMC136150

[143] Gallagher GR Brehm MA Finberg RW Barton BA Shultz LD Greiner DL . Viral infection of engrafted human islets leads to diabetes. Diabetes. (2015) 64:1358–69. doi: 10.2337/db14-1020, PMID: 25392246 PMC4375078

[144] Marroqui L Lopes M dos Santos RS Grieco FA Roivainen M Richardson SJ . Differential cell autonomous responses determine the outcome of coxsackievirus infections in murine pancreatic α and β cells. Elife. (2015) 4:1–23. doi: 10.7554/ELIFE.06990, PMID: 26061776 PMC4480275

[145] Kim KW Ho A Alshabee-Akil A Hardikar AA Kay TWH Rawlinson WD . Coxsackievirus B5 infection induces dysregulation of microRNAs predicted to target known type 1 diabetes risk genes in human pancreatic islets. Diabetes. (2016) 65:996–1003. doi: 10.2337/db15-0956, PMID: 26558682

[146] Colli ML Paula FM Marselli L Marchetti P Roivainen M Eizirik DL . Coxsackievirus B tailors the unfolded protein response to favour viral amplification in pancreatic β cells. J Innate Immun. (2019) 11:375–89. doi: 10.1159/000496034, PMID: 30799417 PMC6738210

[147] Buchacher T Honkimaa A Välikangas T Lietzén N Hirvonen MK Laiho JE . Persistent coxsackievirus B1 infection triggers extensive changes in the transcriptome of human pancreatic ductal cells. iScience. (2022) 25. doi: 10.1016/j.isci.2021.103653, PMID: 35024587 PMC8728469

[148] Sioofy-Khojine AB Richardson SJ Locke JM Oikarinen S Nurminen N Laine AP . Detection of enterovirus RNA in peripheral blood mononuclear cells correlates with the presence of the predisposing allele of the type 1 diabetes risk gene IFIH1 and with disease stage. Diabetologia. (2022) 65:1701–9. doi: 10.1007/S00125-022-05753-Y, PMID: 35867130 PMC9477938

[149] Veronese-Paniagua DA Maestas MM Hernandez-Rincon DC Hinshaw KE Ishahak M Taylor JP . Coxsackievirus B infection invokes unique cell-type-specific responses in primary human pancreatic islets. Cell Rep. (2025) 44:116211. doi: 10.1016/J.CELREP.2025.116211, PMID: 40892543 PMC12655612

[150] Martino TA Petric M Brown M Aitken K Gauntt CJ Richardson CD . Cardiovirulent coxsackieviruses and the decay-accelerating factor (CD55) receptor. Virology. (1998) 244:302–14. doi: 10.1006/VIRO.1998.9122, PMID: 9601501

[151] He Y Chipman PR Howitt J Bator CM Whitt MA Baker TS . Interaction of coxsackievirus B3 with the full length coxsackievirus-adenovirus receptor. Nat Struct Biol. (2001) 8:874–8. doi: 10.1038/NSB1001-874, PMID: 11573093 PMC4152846

[152] Kallewaard NL Zhang L Chen JW Guttenberg M Sanchez MD Bergelson JM . Tissue-specific deletion of the coxsackievirus and adenovirus receptor protects mice from virus-induced pancreatitis and myocarditis. Cell Host Microbe. (2009) 6:91–8. doi: 10.1016/j.chom.2009.05.018, PMID: 19616768 PMC2761025

[153] Bergelson JM Coyne CB . Picornavirus entry. In: Viral Entry into Host Cells New York, NY: Springer (2013). p. 24–41. 10.1007/978-1-4614-7651-1_2 23884584

[154] Wang Q Yang Q Liu C Wang G Song H Shang G . Molecular basis of differential receptor usage for naturally occurring CD55-binding and -nonbinding coxsackievirus B3 strains. Proc Natl Acad Sci U.S.A. (2022) 119:e2118590119. doi: 10.1073/PNAS.2118590119, PMID: 35046043 PMC8794823

[155] Baggen J Thibaut HJ Strating JRPM Van Kuppeveld FJM . The life cycle of non-polio enteroviruses and how to target it. Nat Rev Microbiol. (2018) 16:368–81. doi: 10.1038/s41579-018-0005-4, PMID: 29626210

[156] Hühn MH McCartney SA Lind K Svedin E Colonna M Flodström-Tullberg M . Melanoma differentiation-associated protein-5 (MDA-5) limits early viral replication but is not essential for the induction of type 1 interferons after Coxsackievirus infection. Virology. (2010) 401:42–8. doi: 10.1016/J.VIROL.2010.02.010, PMID: 20206372

[157] Mukherjee A Morosky SA Delorme-Axford E Dybdahl-Sissoko N Oberste MS Wang T . The coxsackievirus B 3Cpro protease cleaves MAVS and TRIF to attenuate host type I interferon and apoptotic signaling. PloS Pathog. (2011) 7:e1001311. doi: 10.1371/JOURNAL.PPAT.1001311, PMID: 21436888 PMC3059221

[158] Cornell CT Kiosses WB Harkins S Whitton JL . Coxsackievirus B3 proteins directionally complement each other to downregulate surface major histocompatibility complex class I. J Virol. (2007) 81:6785–97. doi: 10.1128/jvi.00198-07, PMID: 17442717 PMC1933326

[159] Vecchio F Carré A Korenkov D Zhou Z Apaolaza P Tuomela S . Coxsackievirus infection induces direct pancreatic β cell killing but poor antiviral CD8+ T cell responses. Sci Adv. (2024) 10. doi: 10.1126/SCIADV.ADL1122, PMID: 38446892 PMC10917340

[160] Wong J Zhang J Si X Gao G Mao I McManus BM . Autophagosome supports coxsackievirus B3 replication in host cells. J Virol. (2008) 82:9143–53. doi: 10.1128/JVI.00641-08, PMID: 18596087 PMC2546883

[161] Kemball CC Alirezaei M Flynn CT Wood MR Harkins S Kiosses WB . Coxsackievirus infection induces autophagy-like vesicles and megaphagosomes in pancreatic acinar cells *In Vivo*. J Virol. (2010) 84:12110–24. doi: 10.1128/JVI.01417-10, PMID: 20861268 PMC2976412

[162] Robinson SM Tsueng G Sin J Mangale V Rahawi S . Coxsackievirus B exits the host cell in shed microvesicles displaying autophagosomal markers. PloS Pathog. (2014) 10:1004045. doi: 10.1371/journal.ppat.1004045, PMID: 24722773 PMC3983045

[163] Chen YH Du W Hagemeijer MC Takvorian PM Pau C Cali A . Phosphatidylserine vesicles enable efficient en bloc transmission of enteroviruses. Cell. (2015) 160:619–30. doi: 10.1016/J.CELL.2015.01.032, PMID: 25679758 PMC6704014

[164] Fu Y Xiong S . Exosomes mediate Coxsackievirus B3 transmission and expand the viral tropism. PloS Pathog. (2023) 19:e1011090. doi: 10.1371/journal.ppat.1011090, PMID: 36634130 PMC9888687

[165] Sin J McIntyre L Stotland A Feuer R Gottlieb RA . Coxsackievirus B escapes the infected cell in ejected mitophagosomes. J Virol. (2017) 91:e01347–17. doi: 10.1128/JVI.01347-17, PMID: 28978702 PMC5709598

[166] Alirezaei M Flynn CT Wood MR Whitton JL . Pancreatic acinar cell-specific autophagy disruption reduces coxsackievirus replication and pathogenesis *in vivo*. Cell Host Microbe. (2012) 11:298–305. doi: 10.1016/J.CHOM.2012.01.014, PMID: 22423969 PMC3308121

[167] Shi J Wong J Piesik P Fung G Zhang J Jagdeo J . Cleavage of sequestosome 1/p62 by an enteroviral protease results in disrupted selective autophagy and impaired NFKB signaling. Basic Res PaPeR Autophagy. (2013) 9:1591–603. doi: 10.4161/auto.26059, PMID: 23989536

[168] Krogvold L Mynarek IM Ponzi E Mørk FB Hessel TW Roald T . Pleconaril and ribavirin in new-onset type 1 diabetes: a phase 2 randomized trial. Nat Med. (2023) 29:2902–8. doi: 10.1038/s41591-023-02576-1, PMID: 37789144 PMC10667091

[169] Oikarinen S Krogvold L Edwin B Buanes T Korsgren O Laiho JE . Characterisation of enterovirus RNA detected in the pancreas and other specimens of live patients with newly diagnosed type 1 diabetes in the DiViD study. Diabetologia. (2021) 64:2491–501. doi: 10.1007/S00125-021-05525-0, PMID: 34390364 PMC8494699

[170] Krogvold L Genoni A Puggioni A Campani D Richardson SJ Flaxman CS . Live enteroviruses, but not other viruses, detected in human pancreas at the onset of type 1 diabetes in the DiViD study. Diabetologia. (2022) 65:2108–20. doi: 10.1007/S00125-022-05779-2, PMID: 35953727 PMC9630231

[171] Krogvold L Leete P Mynarek IM Russell MA Gerling IC Lenchik NI . Detection of antiviral tissue responses and increased cell stress in the pancreatic islets of newly diagnosed type 1 diabetes patients: results from the diViD study. Front Endocrinol (Lausanne). (2022) 13:881997. doi: 10.3389/FENDO.2022.881997, PMID: 35957810 PMC9360491

[172] Lundberg M Krogvold L Kuric E Dahl-JØrgensen K Skog O . Expression of interferon-stimulated genes in insulitic pancreatic islets of patients recently diagnosed with type 1 diabetes. Diabetes. (2016) 65:3104–10. doi: 10.2337/DB16-0616, PMID: 27422384

[173] Buschard K Jensen MH Krogvold L Gerling IC Dahl-Jørgensen K Pedersen K . Type 1 diabetes could begin with alterations in innate anti-viral immunity, which are already at this stage associated with HLA risk haplotypes. Diabetes Metab Res Rev. (2023) 39:e3678. doi: 10.1002/DMRR.3678, PMID: 37395313

[174] Geravandi S Richardson S Pugliese A Maedler K . Localization of enteroviral RNA within the pancreas in donors with T1D and T1D-associated autoantibodies. Cell Rep Med. (2021) 2:100371. doi: 10.1016/j.xcrm.2021.100371, PMID: 34467248 PMC8385321

[175] Hodik M Anagandula M Fuxe J Krogvold L Dahl-Jørgensen K Hyöty H . Coxsackie–adenovirus receptor expression is enhanced in pancreas from patients with type 1 diabetes. BMJ Open Diabetes Res Care. (2016) 4:219. doi: 10.1136/BMJDRC-2016-000219, PMID: 27933184 PMC5129002

[176] Ifie E Russell MA Dhayal S Leete P Sebastiani G Nigi L . Unexpected subcellular distribution of a specific isoform of the Coxsackie and adenovirus receptor, CAR-SIV, in human pancreatic beta cells. Diabetologia. (2018) 61:2344–55. doi: 10.1007/S00125-018-4704-1, PMID: 30074059 PMC6182664

[177] Tegehall A Ingvast S Krogvold L Dahl-Jørgensen K Korsgren O . Reduced expression of central innate defense molecules in pancreatic biopsies from subjects with Type 1 diabetes. Acta Diabetol. (2024) 61:1117–27. doi: 10.1007/S00592-024-02286-1, PMID: 38717484 PMC11379773

[178] Von Herrath M Sanda S Herold K . Type 1 diabetes as a relapsing-remitting disease? Nat Rev Immunol. (2007) 7:988–94. doi: 10.1038/nri2192, PMID: 17982429

[179] Thielen L Shalev A . Diabetes pathogenic mechanisms and potential new therapies based upon a novel target called TXNIP. Curr Opin Endocrinol Diabetes Obes. (2018) 25:75–80. doi: 10.1097/MED.0000000000000391, PMID: 29356688 PMC5831522

[180] Minn AH Hafele C Shalev A . Thioredoxin-interacting protein is stimulated by glucose through a carbohydrate response element and induces β-cell apoptosis. Endocrinology. (2005) 146:2397–405. doi: 10.1210/en.2004-1378, PMID: 15705778

[181] Saxena G Chen J Shalev A . Intracellular shuttling and mitochondrial function of thioredoxin- interacting protein. J Biol Chem. (2010) 285:3997–4005. doi: 10.1074/jbc.M109.034421, PMID: 19959470 PMC2823541

[182] Filios SR Xu G Chen J Hong K Jing G Shalev A . MicroRNA-200 is induced by thioredoxin-interacting protein and regulates Zeb1 protein signaling and beta cell. J Biol Chem. (2014) 289:36275–83. doi: 10.1074/jbc.M114.592360, PMID: 25391656 PMC4276888

[183] Harmon JS Stein R Robertson RP . Oxidative stress-mediated, post-translational loss of MafA protein as a contributing mechanism to loss of insulin gene expression in glucotoxic beta cells. J Biol Chem. (2005) 280:11107–13. doi: 10.1074/jbc.M410345200, PMID: 15664999

[184] Zhong J Rao X Xu JF Yang P Wang CY . The role of endoplasmic reticulum stress in autoimmune-mediated beta-cell destruction in type 1 diabetes. Exp Diabetes Res. (2012) 2012. doi: 10.1155/2012/238980, PMID: 22454627 PMC3290823

[185] Yamada T Ishihara H Tamura A Takahashi R Yamaguchi S Takei D . WFS1-deficiency increases endoplasmic reticulum stress, impairs cell cycle progression and triggers the apoptotic pathway specifically in pancreatic β-cells. Hum Mol Genet. (2006) 15:1600–9. doi: 10.1093/hmg/ddl081, PMID: 16571599

[186] Özcan U Cao Q Yilmaz E Lee AH Iwakoshi NN Özdelen E . Endoplasmic reticulum stress links obesity, insulin action, and type 2 diabetes. Sci (1979). (2004) 306:457–61. doi: 10.1126/science.1103160, PMID: 15486293

[187] Seo HY Yong DK Lee KM Min AK Kim MK Kim HS . Endoplasmic reticulum stress-induced activation of activating transcription factor 6 decreases insulin gene expression via up-regulation of orphan nuclear receptor small heterodimer partner. Endocrinology. (2008) 149:3832–41. doi: 10.1210/en.2008-0015, PMID: 18450959 PMC2488228

[188] Fonseca SG Gromada J Urano F . Endoplasmic reticulum stress and pancreatic β-cell death. Trends Endocrinol Metab. (2011) 22:266–74. doi: 10.1016/j.tem.2011.02.008, PMID: 21458293 PMC3130122

[189] Eizirik DL Sammeth M Bouckenooghe T Bottu G Sisino G Igoillo-Esteve M . The human pancreatic islet transcriptome: expression of candidate genes for type 1 diabetes and the impact of pro-inflammatory cytokines. PloS Genet. (2012) 8:e1002552. doi: 10.1371/JOURNAL.PGEN.1002552, PMID: 22412385 PMC3297576

[190] Bergholdt R Brorsson C Palleja A Berchtold LA Fløyel T Bang-Berthelsen CH . Identification of novel type 1 diabetes candidate genes by integrating genome-wide association data, protein-protein interactions, and human pancreatic islet gene expression. Diabetes. (2012) 61:954–62. doi: 10.2337/DB11-1263, PMID: 22344559 PMC3314366

[191] Sarugeri E Dozio N Meschi F Pastore MR Bonifacio E . T cell responses to type 1 diabetes related peptides sharing homologous regions. J Mol Med. (2001) 79:213–20. doi: 10.1007/s001090100194, PMID: 11409713

[192] Ashton MP Eugster A Walther D Daehling N Riethausen S Kuehn D . Incomplete immune response to coxsackie B viruses associates with early autoimmunity against insulin. Sci Rep. (2016) 6. doi: 10.1038/srep32899, PMID: 27604323 PMC5015062

[193] Kramná L Kolářová K Oikarinen S Pursiheimo JP Ilonen J Simell O . Gut virome sequencing in children with early islet autoimmunity. Diabetes Care. (2015) 38:930–3. doi: 10.2337/DC14-2490, PMID: 25678103

[194] Zhao G Vatanen T Droit L Park A Kostic AD Poon TW . Intestinal virome changes precede autoimmunity in type I diabetes-susceptible children. Proc Natl Acad Sci U.S.A. (2017) 114:E6166–75. doi: 10.1073/PNAS.1706359114, PMID: 28696303 PMC5544325

[195] Cinek O Kramna L Odeh R Alassaf A Ibekwe MAU Ahmadov G . Eukaryotic viruses in the fecal virome at the onset of type 1 diabetes: A study from four geographically distant African and Asian countries. Pediatr Diabetes. (2021) 22:558–66. doi: 10.1111/PEDI.13207, PMID: 33786936

[196] Green J Casabonne D Newton R . Coxsackie B virus serology and Type 1 diabetes mellitus: A sytematic review of published case-control studies. Diabetic Med. (2004) 21:507–14. doi: 10.1111/J.1464-5491.2004.01182.X, PMID: 15154932

[197] Lee HS Briese T Winkler C Rewers M Bonifacio E Hyoty H . Next-generation sequencing for viruses in children with rapid-onset type 1 diabetes. Diabetologia. (2013) 56:1705–11. doi: 10.1007/S00125-013-2924-Y, PMID: 23657799 PMC4019381

[198] Mercalli A Lampasona V Klingel K Albarello L Lombardoni C Ekström J . No evidence of enteroviruses in the intestine of patients with type 1 diabetes. Diabetologia. (2012) 55:2479–88. doi: 10.1007/S00125-012-2591-4, PMID: 22684312

[199] Isaacs SR Roy A Dance B Ward EJ Foskett DB Maxwell AJ . Enteroviruses and risk of islet autoimmunity or type 1 diabetes: systematic review and meta-analysis of controlled observational studies detecting viral nucleic acids and proteins. Lancet Diabetes Endocrinol. (2023) 11:578–92. doi: 10.1016/S2213-8587(23)00122-5, PMID: 37390839

[200] Baron M Veres A Wolock SL Faust AL Gaujoux R Vetere A . A single-cell transcriptomic map of the human and mouse pancreas reveals inter- and intra-cell population structure. Cell Syst. (2016) 3:346–60. doi: 10.1016/j.cels.2016.08.011, PMID: 27667365 PMC5228327

[201] Steiner DJ Kim A Miller K Hara M . Pancreatic islet plasticity: Interspecies comparison of islet architecture and composition. Islets. (2010) 2:135–45. doi: 10.4161/isl.2.3.11815, PMID: 20657742 PMC2908252

[202] Maestas MM Bui MH Millman JR . Recent progress in modeling and treating diabetes using stem cell-derived islets. Stem Cells Transl Med. (2024) 13:949–58. doi: 10.1093/STCLTM/SZAE059, PMID: 39159002 PMC11465181

[203] Hogrebe NJ Augsornworawat P Maxwell KG Velazco-Cruz L Millman JR . Targeting the cytoskeleton to direct pancreatic differentiation of human pluripotent stem cells. Nat Biotechnol. (2020) 38:460–70. doi: 10.1038/s41587-020-0430-6, PMID: 32094658 PMC7274216

[204] Hogrebe NJ Maxwell KG Augsornworawat P Millman JR . Generation of insulin-producing pancreatic β cells from multiple human stem cell lines. Nat Protoc. (2021) 16:4109. doi: 10.1038/S41596-021-00560-Y, PMID: 34349281 PMC8529911

[205] Pagliuca FW Millman JR Gürtler M Segel M Van Dervort A Ryu JH . Generation of functional human pancreatic β cells *in vitro*. Cell. (2014) 159:428–39. doi: 10.1016/j.cell.2014.09.040, PMID: 25303535 PMC4617632

[206] Rezania A Bruin JE Arora P Rubin A Batushansky I Asadi A . Reversal of diabetes with insulin-producing cells derived *in vitro* from human pluripotent stem cells. Nat Biotechnol. (2014) 32:1121–33. doi: 10.1038/nbt.3033, PMID: 25211370

[207] Velazco-Cruz L Song J Maxwell KG Goedegebuure MM Augsornworawat P Hogrebe NJ . Acquisition of dynamic function in human stem cell-derived β Cells. Stem Cell Rep. (2019) 12:351–65. doi: 10.1016/j.stemcr.2018.12.012, PMID: 30661993 PMC6372986

[208] Nair GG Liu JS Russ HA Tran S Saxton MS Chen R . Recapitulating endocrine cell clustering in culture promotes maturation of human stem-cell-derived β cells. Nat Cell Biol. (2019) 21:263–74. doi: 10.1038/s41556-018-0271-4, PMID: 30710150 PMC6746427

[209] Veres A Faust AL Bushnell HL Engquist EN Kenty JHR Harb G . Charting cellular identity during human *in vitro* β-cell differentiation. Nature. (2019) 569:368–73. doi: 10.1038/s41586-019-1168-5, PMID: 31068696 PMC6903417

[210] Alvarez-Dominguez JR Donaghey J Rasouli N Kenty JHR Helman A Charlton J . Circadian entrainment triggers maturation of human *in vitro* islets. Cell Stem Cell. (2020) 26:108–122.e10. doi: 10.1016/J.STEM.2019.11.011, PMID: 31839570

[211] Velazco-Cruz L Goedegebuure MM Millman JR . Advances toward engineering functionally mature human pluripotent stem cell-derived β Cells. Front Bioeng Biotechnol. (2020) 8:786. doi: 10.3389/FBIOE.2020.00786, PMID: 32733873 PMC7363766

[212] Hogrebe NJ Ishahak M Millman JR . Developments in stem cell-derived islet replacement therapy for treating type 1 diabetes. Cell Stem Cell. (2023) 30:530–48. doi: 10.1016/j.stem.2023.04.002, PMID: 37146579 PMC10167558

[213] Augsornworawat P Maxwell KG Velazco-Cruz L Millman JR . Single-cell transcriptome profiling reveals β Cell maturation in stem cell-derived islets after transplantation. Cell Rep. (2020) 30:530–48. doi: 10.1016/j.celrep.2020.108067, PMID: 32846125 PMC7491368

[214] Augsornworawat P Millman JR . Single-cell RNA sequencing for engineering and studying human islets. Curr Opin BioMed Eng. (2020) 16:27–33. doi: 10.1016/J.COBME.2020.06.003, PMID: 33738370 PMC7963276

[215] Augsornworawat P Hogrebe NJ Ishahak M Schmidt MD Marquez E Maestas MM . Single-nucleus multi-omics of human stem cell-derived islets identifies deficiencies in lineage specification. Nat Cell Biol. (2023) 25:904. doi: 10.1038/S41556-023-01150-8, PMID: 37188763 PMC10264244

[216] Zhu H Wang G Nguyen-Ngoc KV Kim D Miller M Goss G . Understanding cell fate acquisition in stem cell-derived pancreatic islets using single-cell multiome-inferred regulomes. Dev Cell. (2023) 58:727. doi: 10.1016/J.DEVCEL.2023.03.011, PMID: 37040771 PMC10175223

[217] Weng C Xi J Li H Cui J Gu A Lai S . Single-cell lineage analysis reveals extensive multimodal transcriptional control during directed beta-cell differentiation. Nat Metab. (2020) 2:1443–58. doi: 10.1038/s42255-020-00314-2, PMID: 33257854 PMC7744443

[218] Schmidt MD Ishahak M Augsornworawat P Millman JR . Comparative and integrative single cell analysis reveals new insights into the transcriptional immaturity of stem cell-derived β cells. BMC Genomics. (2024) 25. doi: 10.1186/S12864-024-10013-X, PMID: 38267908 PMC10807170

[219] Millman JR Xie C Van Dervort A Gürtler M Pagliuca FW Melton DA . Generation of stem cell-derived β-cells from patients with type 1 diabetes. Nat Commun. (2016) 7. doi: 10.1038/ncomms11463, PMID: 27163171 PMC4866045

[220] Millman JR Pagliuca FW . Autologous pluripotent stem cell-derived β-like cells for diabetes cellular therapy. Diabetes. (2017) 66:1111–20. doi: 10.2337/db16-1406, PMID: 28507211

[221] Rabhi N Salas E Froguel P Annicotte JS . Role of the unfolded protein response in β cell compensation and failure during diabetes. J Diabetes Res. (2014) 2014:795171. doi: 10.1155/2014/795171, PMID: 24812634 PMC4000654

[222] Maxwell KG Augsornworawat P Velazco-Cruz L Kim MH Asada R Hogrebe NJ . Gene-edited human stem cell–derived β cells from a patient with monogenic diabetes reverse preexisting diabetes in mice. Sci Transl Med. (2020) 12(540). doi: 10.1126/scitranslmed.aax9106, PMID: 32321868 PMC7233417

[223] Kitamura RA Maxwell KG Ye W Kries K Brown CM Augsornworawat P . Multidimensional analysis and therapeutic development using patient iPSC–derived disease models of Wolfram syndrome. JCI Insight. (2022) 7. doi: 10.1172/JCI.INSIGHT.156549, PMID: 36134655 PMC9675478

[224] Amylyx Pharmaceuticals Announces Interim Data From Ongoing Phase 2 HELIOS Clinical Trial Demonstrating Improvements in Pancreatic Function and Glycemic Control with AMX0035 in People with Wolfram Syndrome | Amylyx. Available online at: https://www.amylyx.com/news/amylyx-pharmaceuticals-announces-interim-data-from-ongoing-phase-2-helios-clinical-trial-demonstrating-improvements-in-pancreatic-function-and-glycemic-control-with-amx0035-in-people-with-wolfram-syndrome (Accessed August 17, 2025).

[225] Maxwell KG Millman JR . Applications of iPSC-derived beta cells from patients with diabetes. Cell Rep Med. (2021) 2:100238. doi: 10.1016/j.xcrm.2021.100238, PMID: 33948571 PMC8080107

[226] Maestas MM Ishahak M Augsornworawat P Veronese-Paniagua DA Maxwell KG Velazco-Cruz L . Identification of unique cell type responses in pancreatic islets to stress. Nat Commun. (2024) 15. doi: 10.1038/S41467-024-49724-W, PMID: 38956087 PMC11220140

[227] D’Oliveira Albanus R Zhang X Zhao Z Taylor HJ Tang X Han Y . Integrative single-cell multi-omics profiling of human pancreatic islets identifies T1D-associated genes and regulatory signals. Cell Rep. (2025) 44. doi: 10.1016/j.celrep.2025.116065, PMID: 40737125 PMC12477748

[228] Balboa D Saarimäki-Vire J Borshagovski D Survila M Lindholm P Galli E . Insulin mutations impair beta-cell development in a patient-derived iPSC model of neonatal diabetes. Elife. (2018) 7. doi: 10.7554/eLife.38519, PMID: 30412052 PMC6294552

[229] Braverman-Gross C Nudel N Ronen D Beer NL McCarty MI Benvenisty N . Derivation and molecular characterization of pancreatic differentiated MODY1-iPSCs. Stem Cell Res. (2018) 31:16–26. doi: 10.1016/J.SCR.2018.06.013, PMID: 29990710

[230] Kahraman S Dirice E Basile G Diegisser D Alam J Johansson BB . Abnormal exocrine–endocrine cell cross-talk promotes β-cell dysfunction and loss in MODY8. Nat Metab. (2022) 4:76–89. doi: 10.1038/s42255-021-00516-2, PMID: 35058633 PMC12706769

[231] Teo AKK Windmueller R Johansson BB Dirice E Njolstad PR Tjora E . Derivation of human induced pluripotent stem cells from patients with maturity onset diabetes of the young. J Biol Chem. (2013) 288:5353–6. doi: 10.1074/jbc.C112.428979, PMID: 23306198 PMC3581399

[232] González BJ Zhao H Niu J Williams DJ Lee J Goulbourne CN . Reduced calcium levels and accumulation of abnormal insulin granules in stem cell models of HNF1A deficiency. Commun Biol. (2022) 5:1–17. doi: 10.1038/s42003-022-03696-z, PMID: 35918471 PMC9345898

[233] Cardenas-Diaz FL Osorio-Quintero C Diaz-Miranda MA Kishore S Leavens K Jobaliya C . Modeling monogenic diabetes using human ESCs reveals developmental and metabolic deficiencies caused by mutations in HNF1A. Cell Stem Cell. (2019) 25:273–289.e5. doi: 10.1016/J.STEM.2019.07.007, PMID: 31374199 PMC6785828

[234] Hermann FM Kjærgaard MF Tian C Tiemann U Jackson A Olsen LR . An insulin hypersecretion phenotype precedes pancreatic β cell failure in MODY3 patient-specific cells. Cell Stem Cell. (2023) 30:38–51.e8. doi: 10.1016/J.STEM.2022.12.001, PMID: 36563694

[235] Cujba AM Alvarez-Fallas ME Pedraza-Arevalo S Laddach A Shepherd MH Hattersley AT . An HNF1α truncation associated with maturity-onset diabetes of the young impairs pancreatic progenitor differentiation by antagonizing HNF1β function. Cell Rep. (2022) 38:110425. doi: 10.1016/J.CELREP.2022.110425, PMID: 35235779 PMC8905088

[236] Chandra V Ibrahim H Halliez C Prasad RB Vecchio F Dwivedi OP . The type 1 diabetes gene TYK2 regulates β-cell development and its responses to interferon-α. Nat Commun. (2022) 13:1–16. doi: 10.1038/s41467-022-34069-z, PMID: 36289205 PMC9606380

[237] Nair AK Traurig M Sutherland JR Muller YL Grellinger ED Saporito L . Generation of Isogenic hiPSCs with Targeted Edits at Multiple Intronic SNPs to Study the Effects of the Type 2 Diabetes Associated KCNQ1 Locus in American Indians. Cells. (2022) 11:1446. doi: 10.3390/CELLS11091446, PMID: 35563754 PMC9102014

[238] Lau HH Krentz NAJ Abaitua F Perez-Alcantara M Chan JW Ajeian J . PAX4 loss of function increases diabetes risk by altering human pancreatic endocrine cell development. Nat Commun. (2023) 14:1–19. doi: 10.1038/s41467-023-41860-z, PMID: 37777536 PMC10542369

[239] Tatovic D Narendran P Dayan CM . A perspective on treating type 1 diabetes mellitus before insulin is needed. Nat Rev Endocrinol. (2023) 19:361–70. doi: 10.1038/s41574-023-00816-5, PMID: 36914759

[240] Evans-Molina C Dor Y Lernmark Å Mathieu C Millman JR Mirmira RG . The heterogeneity of type 1 diabetes: implications for pathogenesis, prevention, and treatment—2024 Diabetes, Diabetes Care, and Diabetologia Expert Forum. Diabetologia. (2025) 68:1859–78. doi: 10.1007/S00125-025-06462-Y, PMID: 40736750 PMC12361346

[241] Evans-Molina C Dor Y Lernmark Å Mathieu C Millman JR Mirmira RG . The heterogeneity of type 1 diabetes: implications for pathogenesis, prevention, and treatment-2024 diabetes, diabetes care, and diabetologia expert forum. Diabetes Care. (2025) 48:1651–67. doi: 10.2337/DCI25-0013, PMID: 40736378 PMC12454688

[242] Evans-Molina C Dor Y Lernmark Å Mathieu C Millman JR Mirmira RG . The heterogeneity of type 1 diabetes: implications for pathogenesis, prevention, and treatment—2024 diabetes, diabetes care, and diabetologia expert forum. Diabetes. (2025) 74:1730–1747. doi: 10.2337/DBI25-0011, PMID: 40736345 PMC12454687

[243] Krischer JP Liu X Vehik K Akolkar B Hagopian WA Rewers MJ . Predicting islet cell autoimmunity and type 1 diabetes: an 8-year TEDDY study progress report. Diabetes Care. (2019) 42:1051–60. doi: 10.2337/DC18-2282, PMID: 30967432 PMC6609953

[244] In’t Veld P . Insulitis in human type 1 diabetes: A comparison between patients and animal models. Semin Immunopathol. (2014) 36:569–79. doi: 10.1007/S00281-014-0438-4, PMID: 25005747 PMC4186970

[245] Munn DH Mellor AL . IDO in the tumor microenvironment: inflammation, counter-regulation, and tolerance. Trends Immunol. (2016) 37:193–207. doi: 10.1016/J.IT.2016.01.002, PMID: 26839260 PMC4916957

[246] Bone RN Oyebamiji O Talware S Selvaraj S Krishnan P Syed F . A computational approach for defining a signature of β-cell golgi stress in diabetes. Diabetes. (2020) 69:2364–76. doi: 10.2337/DB20-0636, PMID: 32820009 PMC7576569

